# Conservation performance of different conservation governance regimes in the Peruvian Amazon

**DOI:** 10.1038/s41598-017-10736-w

**Published:** 2017-09-12

**Authors:** Judith Schleicher, Carlos A. Peres, Tatsuya Amano, William Llactayo, Nigel Leader-Williams

**Affiliations:** 1 Department of Geography, University of Cambridge, Cambridge, CB2 3EN UK; 2 UN Environment World Conservation Monitoring Centre, Cambridge, CB3 0DL UK; 3 School of Environmental Sciences, University of East Anglia, Norwich, NR4 7TJ UK; 4 Conservation Science Group, Department of Zoology, University of Cambridge, Cambridge, CB2 3QZ UK; 5 Centre for the Study of Existential Risk, University of Cambridge, Cambridge, CB2 1SG UK; 6 Dirección General de Ordenamiento Territorial Ambiental, Ministry of Environment, San Isidro, Lima, 27 Peru

## Abstract

State-controlled protected areas (PAs) have dominated conservation strategies globally, yet their performance relative to other governance regimes is rarely assessed comprehensively. Furthermore, performance indicators of forest PAs are typically restricted to deforestation, although the extent of forest degradation is greater. We address these shortfalls through an empirical impact evaluation of state PAs, Indigenous Territories (ITs), and civil society and private Conservation Concessions (CCs) on deforestation and degradation throughout the Peruvian Amazon. We integrated remote-sensing data with environmental and socio-economic datasets, and used propensity-score matching to assess: (i) how deforestation and degradation varied across governance regimes between 2006–2011; (ii) their proximate drivers; and (iii) whether state PAs, CCs and ITs avoided deforestation and degradation compared with logging and mining concessions, and the unprotected landscape. CCs, state PAs, and ITs all avoided deforestation and degradation compared to analogous areas in the unprotected landscape. CCs and ITs were on average more effective in this respect than state PAs, showing that local governance can be equally or more effective than centralized state regimes. However, there were no consistent differences between conservation governance regimes when matched to logging and mining concessions. Future impact assessments would therefore benefit from further disentangling governance regimes across unprotected land.

## Introduction

The rapid conversion and degradation of tropical forests is arguably one of the most significant challenges of contemporary global change. It is of particular concern given the associated loss of biodiversity, ecosystem services and human livelihoods, and in light of international efforts to curb carbon emissions^1–5^. The mainstream global conservation strategy to address these concerns has been the establishment of state-controlled protected areas (PAs)^6, 7^. As a result, the global network of state PAs has expanded rapidly over the past decades, with some 202,467 terrestrial PAs designated so far^7, 8^. Furthermore, governments have agreed to ambitious targets for expanding these further^9^. Despite these considerable conservation efforts, the conversion and degradation of tropical forests continue largely unabated^10–13^. Consequently, calls have mounted for rigorous evaluations of the impacts and effectiveness of different conservation approaches^14–16^. This is especially relevant to forest degradation, as comparatively little research attention has been paid to understanding its proximate drivers and how it can be reduced by conservation interventions. In addition, an extensive literature has highlighted that no single governance regime, such as state PAs, is a silver bullet for sustainable resource use of forests and other common-pool resources (e.g. refs 17–19). Conservationists have therefore sought a diversification of conservation approaches. In particular, attention has been drawn to the potential contributions of formally designated Indigenous Territories (ITs) and civil society and private PAs to tropical forest conservation^20–22^.

Consequently, much debate surrounds the relative impacts of conservation approaches under different governance regimes to meet conservation objectives. Methodological advances have allowed researchers to more confidently attribute observed impacts to specific interventions and eliminate competing explanations^23^. As a result, a small but rapidly increasing number of studies has applied quasi-experimental ‘matching’ approaches, which compare conservation impacts of state PAs with the ‘unprotected’ landscape, while controlling for covariates that are expected to affect habitat conversion probability and PA location (e.g. refs 24–26). However, these studies have mainly focused on measuring the impacts of state PAs on rates of deforestation (e.g. refs 24, 26–29) and fire occurrence^30^. Studies using counterfactual approaches to examine the impacts of PAs on a wider range of human disturbances are still lacking. Furthermore, only few counterfactual studies have assessed the conservation impacts of land-use designations other than state PAs^31–33^, despite an increasing awareness of the importance of other governance types. Even where previous assessments have been undertaken, the surrounding unprotected landscape has been treated as homogenous, without distinguishing between different forms of governance and varying legal restrictions on natural resource extraction (but see refs 31, 34).

**Figure 1:**
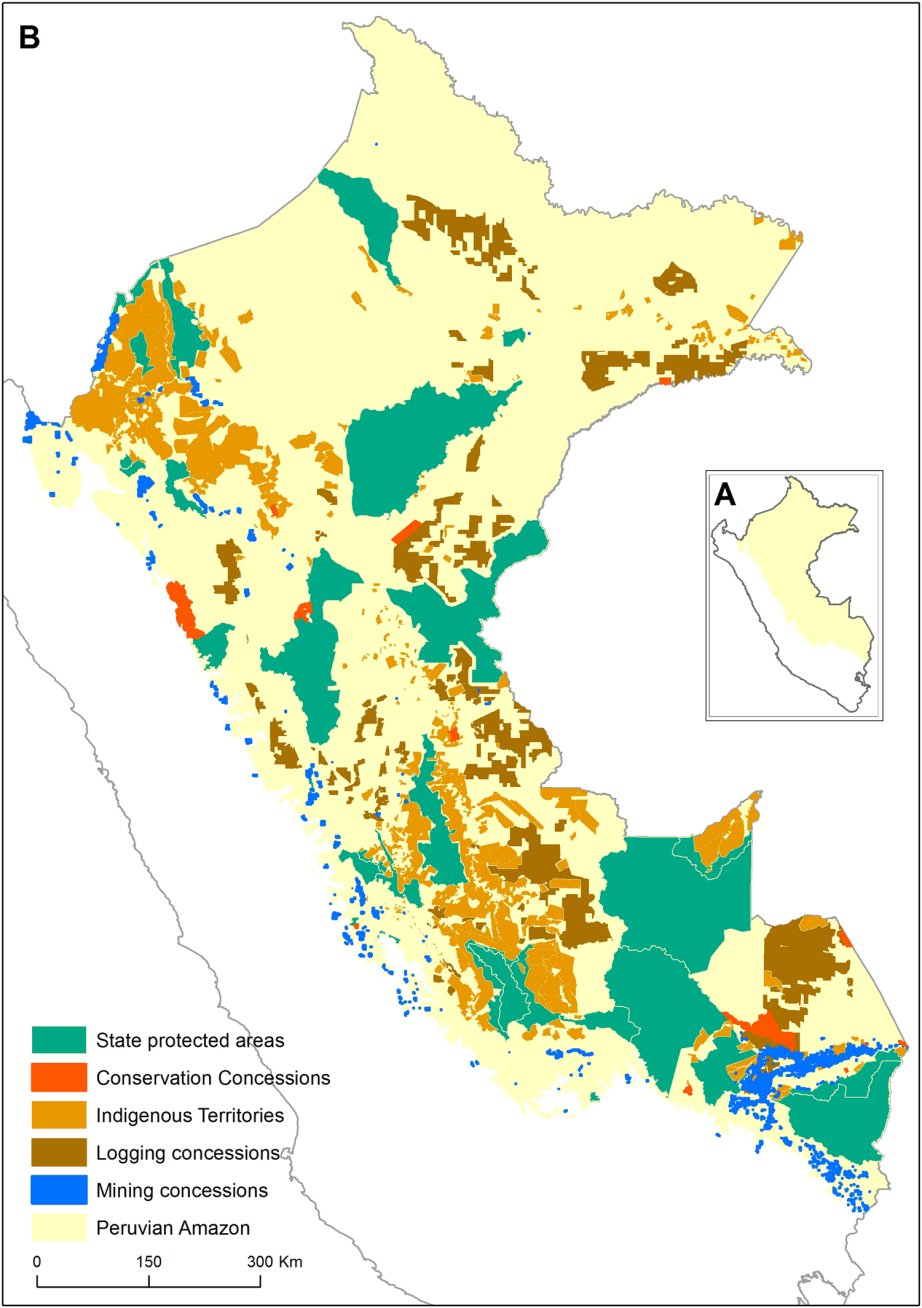
Map of (**A**) Peru and (**B**) the Peruvian Amazon showing the state protected areas (n = 30), Conservation Concessions (n = 13), Indigenous Territories (n = 992), logging concessions (n = 441) and mining concessions (n = 1450) included in the study. The map was produced in ArcMap 10.3 (http://desktop.arcgis.com/en/arcmap/) based on shapefiles collated for this study.

Here, we assess the impacts of three different conservation governance regimes on rates of deforestation (defined as total forest cover loss) and forest degradation (defined as other human-induced forest disturbances, mainly due to selective logging, logging tracks and fire) across the Peruvian Amazon from 2006 to 2011, compared to three alternative forest governance regimes. Peru is an excellent case study as it is very biodiverse and has established a wide range of forest conservation governance regimes with analogues in many other countries. This includes state PAs, ITs and Conservation Concessions (CCs), a novel type of civil society and private PAs, represented in Peru and various other countries^35–37^. Our study has three main objectives: (i) assess how deforestation and forest degradation varied across protected and unprotected forest governance types throughout the Peruvian Amazon; (ii) determine the proximate environmental and socio-economic predictors of deforestation and forest degradation (Supplementary Table S1); and (iii) control for these predictors to assess whether state PAs, ITs and/or CCs have avoided deforestation and forest degradation compared to three unprotected forest governance counterfactuals. These counterfactuals comprise logging concessions, mining concessions and the wider unprotected landscape (defined as the land beyond formally-recognized and nationally-mapped governance regimes) (Fig. 1). Our study area covers 74% of the entire Peruvian Amazon, excluding areas covered by water bodies, clouds and shadows (see Methods).

## Results

### Deforestation and forest degradation across forest governance regimes

Across the entire study area, the rates of forest degradation and deforestation were 0.53% and 0.35%, respectively, between 2006 and 2011. Over that period, forest degradation (193,648 ha) affected an area 47.5% larger than that affected by deforestation (131,320 ha). The spatial distribution of deforestation and forest degradation was highly heterogeneous, as 78% of all deforestation and 66% of forest degradation was concentrated in only three regions of the Peruvian Amazon (see Supplementary Fig. S1).

**Figure 2:**
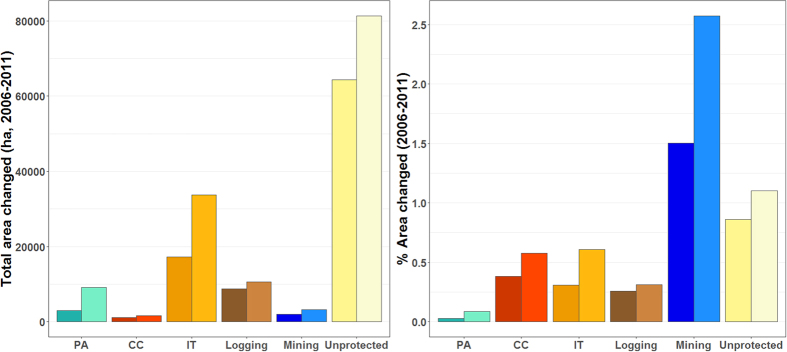
Deforestation (darker colours) and forest degradation (lighter colours) between 2006 and 2011 in terms of the total area affected (left panel) and percentage of area changed (right panel) across the main protected and unprotected forest governance regimes in the Peruvian Amazon: state protected areas (PA, n = 30), Conservation Concessions (CC, n = 13), Indigenous Territories (IT, n = 992), logging (n = 441) and mining (n = 1450) concessions, and the wider unprotected landscape (Unprotected).

Overall rates of deforestation and forest degradation were higher across the wider unprotected landscape than in the other forest governance types combined (Fig. 2). In terms of proportional areas affected, state PAs experienced the lowest deforestation (0.03%) and degradation rates (0.09%). In contrast, mining concessions were most severely affected by deforestation (1.5%) and degradation (2.6%), even more so than the wider unprotected landscape (Fig. 2).

### Drivers of forest degradation and deforestation

The proximate environmental and socio-economic drivers explained 50.6% and 48.4% of the deviance of spatial generalized linear models of forest degradation and deforestation, respectively. For forest degradation, measures of accessibility, especially distance to previously deforested areas and travel time to urban centers, were among the main predictors. Furthermore, forest degradation decreased with distance to settlements and roads, and was lower inside state PAs and ITs (see Supplementary Table S2 for details).

As with forest degradation, accessibility, measured as proximity to previously deforested areas and settlements, was the main predictor of deforestation, in addition to administrative region. However, the latter was a stronger predictor of deforestation than forest degradation. The likelihood of deforestation also decreased inside state PAs and ITs as well as in areas on steeper slopes, farther from roads and urban centres. Whilst logging concessions reduced the likelihood of both deforestation and forest degradation, they contributed little explanatory power to predicting either of them. Forest type and proximity to rivers also did not play a large role in explaining deforestation or forest degradation (see Supplementary Table S2).

However, the relative importance of different administrative regions differed between degradation and deforestation. Furthermore, in contrast to deforestation, elevation rather than slope, and the number of wet months rather than total annual rainfall, were better predictors of the likelihood of forest degradation (see Supplementary Table S2).

### Conservation performance of different forest conservation governance regimes

**Figure 3:**
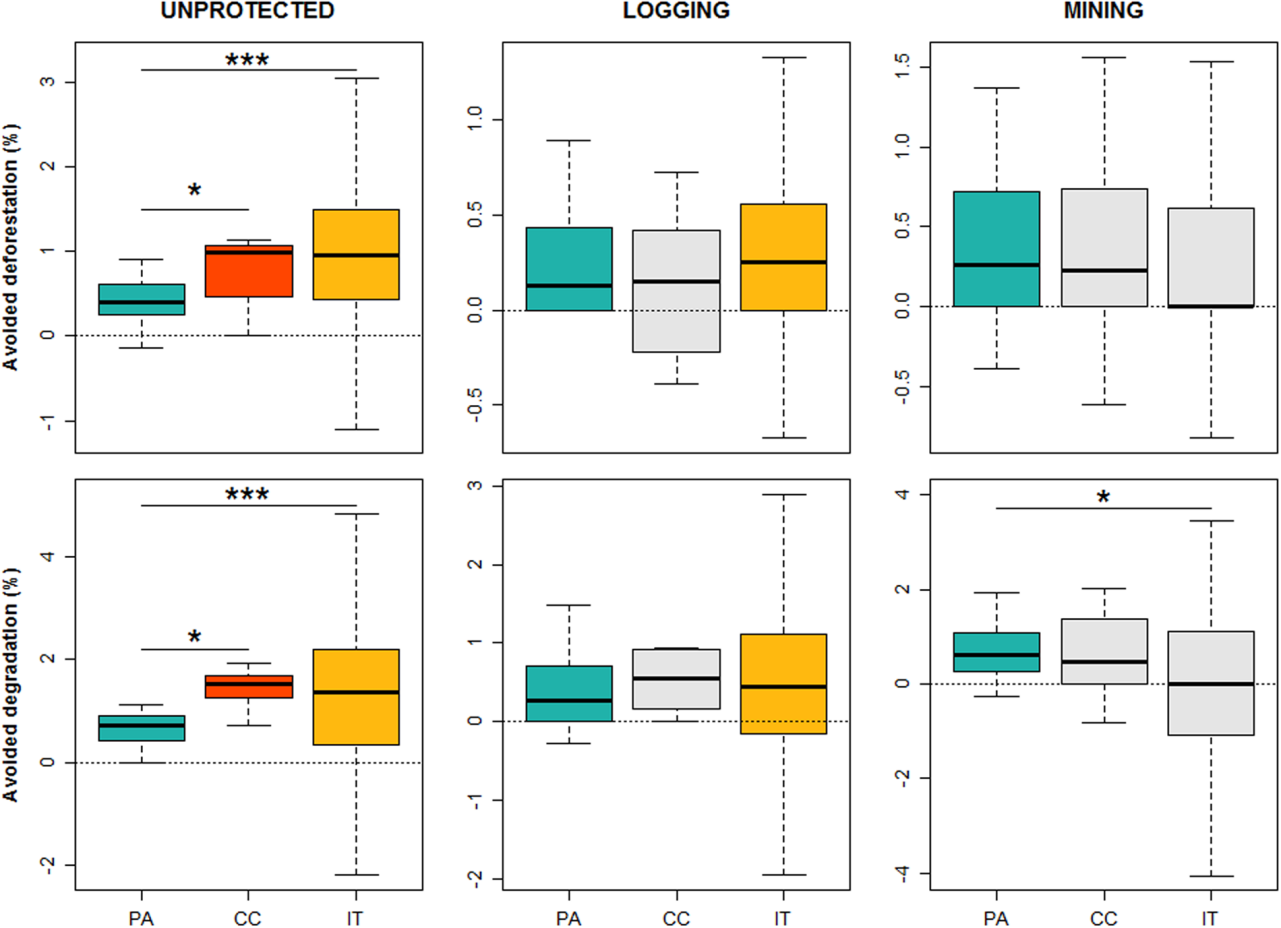
Impacts of different conservation governance regimes on 2006–2011 deforestation (upper panels) and forest degradation rates (lower panels), compared to the wider unprotected matrix (UNPROTECTED), logging and mining concessions based on propensity score matching. Positive effects (above horizontal dashed lines) indicate avoided deforestation or degradation of state ﻿protected areas (PA), Conservation Concessions (CC) and Indigenous Territories (IT) compared to matched controls; where these effects were significantly different from zero (p < 0.05) the boxplots are shown in colour and where not significant the boxplots are shown in grey. Whiskers extend to the most extreme data point which is no more than 1.5 times the interquartile range. Asterisks indicate significant differences between conservation governance regimes (*p < 0.05; ***p < 0.001). Outliers were computed but not included in the boxplots for purposes of clarity.

When matched to the wider unprotected landscape, state PAs, CCs, and ITs exhibited significantly lower deforestation and forest degradation rates (Fig. 3). CCs and ITs exhibited greater variation in terms of their avoided deforestation and degradation than state PAs. Meanwhile, CCs on average avoided twice as much deforestation (median: 0.99%) and forest degradation (1.53%) than state PAs (deforestation: 0.40%; degradation: 0.72%), and slightly more than ITs (deforestation: 0.95%; degradation: 1.37%). These differences were significant between CCs and state PAs, and between ITs and state PAs, but not between CCs and ITs (Fig. 3; Supplementary Table S3). These absolute rates of avoided deforestation and degradation were low due to the relatively low rates of change across the study area (Fig. 2). However, relative to the rates of change in the matched unprotected landscape, the reduction in deforestation and forest degradation was very large across state PAs (median [first quartile, third quartile]: deforestation: 100.0% [88.1%, 100.0%]; degradation: 85.2% [73.3%, 95.2%]), CCs (deforestation: 99.0% [38.5%, 100.0%]; degradation: 91.6% [58.3%, 100.0%]), and ITs (deforestation: 100.0% [60.0%, 100.0%]; degradation: 75.0% [25.0%, 100.0%]).

Compared to matched areas in logging concessions, both state PAs and ITs had significantly lower deforestation and degradation rates, while there was no significant difference for CCs. There were also no significant differences between state PAs, ITs and CCs in terms of avoided deforestation or degradation compared to logging concessions (Fig. 3).

The percentage of data points successfully matched to mining concessions was consistently low, ranging from 11% to 19%, compared to the matching success for logging concessions (24% to 70%) and the wider unprotected landscape (53% to 83%). This was due to mining concessions being located in areas exposed to higher deforestation and forest degradation pressures. This indicates that mining concessions provided a poor comparison for the three conservation governance regimes. In the few areas successfully matched to mining concessions, only state PAs avoided deforestation and forest degradation rates. However, there was no significant difference in avoided deforestation between state PAs, CCs and ITs. In terms of avoided forest degradation, state PAs were significantly more effective than ITs, but there was no significant difference between CCs and state PAs, nor between CCs and ITs (Fig. 3).

For all three conservation strategies, regardless of to which of the three counterfactuals they were matched, the median absolute avoided degradation was consistently higher than avoided deforestation, except for ITs matched to mining concessions, for which both the median avoided deforestation and degradation were equal (Fig. 3).

## Discussion

Tropical forest conversion and degradation have escalated despite the investment of considerable conservation efforts. Nevertheless, the relative performance of different conservation governance types compared to alternative forms of land use has rarely been comprehensively assessed and little attention has been paid to forest degradation. Here we provide one of the first quantitative counterfactual assessments of the impacts of three main conservation governance types — namely state PAs, ITs and CCs — on deforestation and forest degradation across the Peruvian Amazon, comparing disaggregated impacts to logging concessions, mining concessions and the wider unprotected landscape.

### Conservation performance of different forest governance regimes

All three types of conservation governance regimes significantly avoided rates of deforestation and forest degradation compared to analogous areas in the wider unprotected landscape. Nevertheless, none were immune to these anthropogenic impacts. Surprisingly, while rates of deforestation and forest degradation between 2006 and 2011 were higher in CCs and ITs than in state PAs (Fig. 2), these effects were reversed when controlling for location-specific covariates (Fig. 3). Consequently, CCs and ITs were on average more effective than state PAs in avoiding deforestation and forest degradation compared to the wider unprotected landscape. However, state PAs most consistently reduced deforestation and forest degradation, exhibiting less variation across different PAs, regardless of which of the three unprotected land uses they were compared to. These findings therefore substantially add to previous counterfactual matching studies showing that state-controlled PAs are effective at avoiding deforestation compared to unprotected areas^13, 24–27, 38^. In addition to extending this generalization to the Peruvian Amazon^29^, our findings build on previous evidence revealing that ITs are particularly effective at counteracting location-specific deforestation relative to state-controlled PAs in Brazil^26, 28^. To our knowledge, this is the first study to evaluate civil society and private PAs using a counterfactual matching approach. It would be valuable for future research to assess whether the results reported here also apply to data collected post-2011. Our findings add to the growing body of research that local forest governance can be equally or more effective than centralized state regimes. Given these conservation benefits, we advocate for the relevant authorities to address issues related to the designation of CCs and ITs in Peru. These include (i) reducing the complexity and length of the application processes, which often take several years to complete^39^ and (ii) adding to the very few ITs that have been granted to indigenous communities during the last decade. This is particularly important in light of the widespread political interests to resist or reverse the decentralization and devolution of the control over forests and other natural resources^40–42^.

Our regression models showed that active logging concessions earmarked for timber extraction reduced the likelihood of deforestation and forest degradation compared to areas not designated as such, when accounting for a large suite of proximate socio-economic and biophysical covariates. This finding seems surprising as conventional logging practices in Peru are widely regarded as unsustainable^43, 44^, although this would comply with the associated legal obligations, which prohibit deforestation in logging concessions to promote sustainable timber extraction^43, 45, 46^. This also corroborates findings based on remote-sensing data from other tropical countries that logging concessions are (i) less likely to be deforested and degraded than matched areas outside the permanent forest estate (Cameroon:^32^), and (ii) equally effective as state PAs in inhibiting deforestation (Sumatra and Kalimantan:^31, 34^). This has led to calls for designating logging concessions as PAs^31^. However, we warn against interpreting this as proof of effectively protected logging concessions, evidence for which emerges when combined with data from other sources. In Peru, government data and interviews with key informants suggest that this is at least partly due to leakage, whereby logging concession timber permits are widely used to legalize timber harvest in non-authorized areas^39, 47^. This emphasizes the importance of combining remote-sensing with field data to explore causal relationships, triangulate findings between data sources and harness their full potential^48^.

### Beyond deforestation: assessments of forest degradation

We further expand on previous studies by showing that the three modes of conservation governance in the Peruvian Amazon were also effective in avoiding forest degradation, and that mean avoided degradation was more extensive than avoided deforestation where this was the case. This is especially relevant given that the overall area of degraded forest between 2006 and 2011 was 47.5% larger than the area deforested, both of which remained largely concentrated in three key hotspots of deforestation, disturbance and selective logging^49, 50^. Proximate socio-economic and environmental drivers were good predictors of where forest degradation and deforestation occurred, and physical accessibility was the main determinant. Although overall degradation had similar proximate drivers as deforestation, some key differences were evident, particularly concerning administrative regions, climatic variables and topography.

The spatial extent and differential drivers of degradation are particularly important as most previous national and international assessments of conservation impacts and drivers of change have been largely restricted to deforestation, thereby neglecting the much wider spectrum of more cryptic anthropogenic disturbances degrading tropical forests^51–53^. This has occurred despite increasing international attention paid to both deforestation and forest degradation, especially in light of the rapid spread of initiatives to reduce emissions from deforestation and forest degradation (REDD)^41, 54, 55^. Therefore, it would be valuable for future smaller-scale assessments to also account for other potentially important, but less detectable drivers that could not be included in our analysis. These include narrower roads and trails for hunting, logging and the extraction of non-timber forest products.

### Measures of ‘success’ and the heterogeneous unprotected landscape

Importantly, our results emphasize that the degree to which different forest governance regimes effectively preclude deforestation and forest degradation depends on which measures of impacts are being assessed. This applies to the absolute versus relative effect compared to the expected pressure of change^26, 28, 38^, the average versus the most consistent effect, but also against what type of ‘unprotected area’ the impact is being evaluated. While all three conservation strategies were effective at avoiding deforestation and degradation compared to the wider unprotected landscape, the impacts were less consistent when compared to logging and mining concessions. Mining concessions experienced the highest proportion of deforestation and forest degradation of all land use designations assessed, as might be expected in light of the recent surge in highly destructive gold mining exploitation in the southern Peruvian Amazon^56, 57^. They were also located in more accessible areas subject to higher human pressures. In turn, this allowed few successful matches and consequently, mining concessions provided poor comparisons for the three conservation strategies.

Differentiating between logging and mining concessions and the wider unprotected landscape is particularly important as nearly all previous matching studies have treated unprotected areas as homogenous, by failing to distinguish between the various types of governance and land-use regulations across these areas (but see refs 31, 34). Here we show that differentiating between these governance arrangements matters when assessing conservation impacts. Therefore our study provides a more nuanced understanding of conservation impacts of different forest governance regimes. Future assessments could benefit from further disentangling land use restrictions and *in situ* governance beyond PA and IT boundaries.

In conclusion, we found that both forest degradation and deforestation exhibited similar proximate drivers throughout the Peruvian Amazon. Accounting for these drivers, state PAs, CCs and ITs reduced the likelihood of deforestation and forest degradation compared to the wider unprotected matrix. These positive impacts are expected to increase as development frontiers expand further into the Peruvian Amazon. By contrast, their impacts were more variable compared to analogous areas within logging and mining concessions. This study therefore illustrates that the type of land use restrictions and governance arrangements in the landscape beyond PA boundaries matter when determining the impacts of different conservation interventions and governance regimes, and therefore warrant more attention in future conservation assessments. Furthermore, given the benefits that local forest governance through CCs and ITs can provide to avoiding deforestation and degradation, we recommend that the government or non-governmental sector should (i) develop policies that facilitate the application process and designation of CCs and ITs, (ii) provide support mechanisms for implementing them, and (iii) assess the factors that contribute to, or inhibit, the effective implementation of CCs, ITs and PAs.

## Methods

### Study area: protected and unprotected forest governance regimes

For this study, we considered the entire Peruvian Amazon as defined by the Peruvian Ministry of Environment (MINAM), which includes both lowland and Andean areas (Fig. 1). Our study area comprises the part of the Peruvian Amazon assessed by our deforestation and forest degradation analysis (see next section and Supplementary Fig. S2). We divided the Peruvian Amazon into forest governance regimes associated with protection and unprotected status based on official land use categories designated by the government. As protected land use designations, we considered the most numerous categories found throughout the Peruvian Amazon where commercial selective logging and deforestation are prohibited, namely national state PAs, CCs and ITs. We included ITs as a protected governance regime^58, 59^ as they have been shown to protect ecosystems in other countries, even if they were established for purposes other than environmental conservation^20^. While state PAs and ITs are owned and managed by the state and indigenous communities, respectively, CCs comprise public land managed by private and civil society actors (e.g. individuals, NGOs, companies) for conservation purposes for a specific period of time. In Peru, CCs are renewable on a 40-year basis and have increasingly spread as a conservation tool across the Peruvian Amazon^60^. CCs have been promoted in numerous countries, including in East Asia, and South and North America^35–37^. We considered state PAs, CCs and ITs designated in or before 2006 throughout the study area (see Fig. 1). This comprised 30 state PAs and 13 CCs. As ITs were very numerous (n > 1400), we only included those with at least 10% forest cover in 2006 and at least 10% of their area found within the study area (n = 992). For the unprotected forest governance regimes, we considered extractive land use designations, namely active logging and mining concessions, as well as the wider unprotected landscape beyond these designations. Logging and mining concessions are granted to non-state actors for the extraction of timber and below-ground minerals, respectively, while the wider unprotected landscape is mainly under state jurisdiction.

### Assessing deforestation and forest degradation

We used the CLASlite system^61^ to map 2006 forest cover and 2006-2011 deforestation and forest degradation based on orthorectified Landsat 5 images (~30 m resolution), obtained from USGS (www.earthexplorer.usgs.org). We created mosaics of 92 L1T-processed Landsat images from 25 Landsat tiles covering over 74% (582,966 km^2^) of the Peruvian Amazon (784,692 km^2^; Supplementary Fig. S2), selected based on the following criteria: (i) coverage of all national state PAs and CCs in place throughout 2007 and 2011; (ii) coverage of the majority of ITs and logging concessions; and (iii) images were preferentially taken in the same month for both years of the analysis. Due to persistent cloud cover over parts of the study area, the majority but not all of the images were taken in 2006 and 2011 (Supplementary Table S4). Evaluating forest cover change based on composite images spanning several years is commonly done (e.g. refs 62, 63). To ensure this did not bias the results, we inspected data layers visually to ensure that the covariate datasets were not systematically evaluated over different time periods.

CLASlite is particularly well suited for the purpose of this study because it (i) was specifically designed to detect deforestation and forest degradation in tropical forests, (ii) has been extensively validated, especially in the Peruvian and Brazilian Amazon^49, 61, 64^, and (iii) has resulted in higher levels of accuracy^65^ than a supervised classification and recent global forest change dataset^11^. CLASlite includes a series of processes that (i) performs radiometric calibration and atmospheric correction on the raw Landsat images, and masks clouds, shadows and water to be excluded from the analysis; (ii) decomposes the remaining pixels into fractional cover classes of photosynthetic vegetation, non-photosynthetic vegetation and bare substrates, through comparison of the spectral signatures of bands 4–7 of each pixel with the spectral signature libraries obtained from extensive field surveys; and (iii) classifies the resulting fractional cover images into forest cover, deforestation and forest degradation (see refs 49, 61 and Supplementary Methods for details). In cases where the automatic masking of cloud, shadow and/or water was insufficient, we carried out additional masking manually in ENVI 4.5.

After image processing in CLASlite, we performed extensive (pixel by pixel) manual post-processing of the 2006 forest cover and the two change detection maps in ENVI 4.5, comprising three components: (i) review of output images by in-country experts from regional and national Peruvian organisations; (ii) visual comparison of the output images with the Landsat input images; and (iii) where required and available, visual comparison of output images with higher resolution Google Earth images. This was done to rectify errors especially due to the following: (i) water, clouds, and shadows that had been missed during the masking process; (ii) natural hydrological changes; (iii) topography; (iv) natural phenological changes; and (v) different forest types, especially bamboo stands. The analysis was validated combining 90 high-resolution RapidEye satellite images (5 m resolution, 25 by 25 km) and extensive field surveys, selecting control points through stratified random sampling^66, 67^. This yielded a 98.1% overall accuracy across the study area based on RapidEye images and an 85.5% accuracy in relatively accessible areas for field surveys (see Supplementary Methods and Tables S4–S5 for details). These levels of accuracy are very similar to previous studies^49, 61, 64^.

### Assessing drivers of forest degradation and deforestation

We combined the resulting deforestation and forest degradation datasets with spatially-explicit environmental and socio-economic data to determine the proximate drivers of deforestation and forest degradation using logistic generalized linear models (GLMs), assuming a binomial distribution and logit link function in the *glm* package in R 3.0.3^68^. The response variables were whether deforestation and forest degradation respectively occurred or not in each pixel. Following previous studies (e.g. refs 24, 30, 62, 69) and based on our own knowledge of the national context, we selected potential predictor variables based on *a priori* expectations of having an effect on deforestation and/or forest degradation. More specifically, we selected measures of human pressure (i.e. distance to settlements, travel time to cities^70^, human population density), access to the forest (i.e. distance to roads, rivers and previous deforestation, topography^71^), suitability for conversion, particularly for agriculture (i.e. slope, elevation, rainfall^72^, number of wet months, forest types), the political and economic environment (i.e. administrative region), and forest governance types with large enough samples (i.e. state PAs, ITs and logging concessions; see Supplementary Table S1 for details). It was not possible to include soil characteristics, agricultural plots and agricultural suitability directly into the models as no relevant data existed for Peru at the national level. We obtained boundaries of national state PAs from MINAM and WWF Peru, boundaries of ITs from the Instituto del Bien Común, boundaries of CCs from the Ministry of Agriculture (MINAG) and regional government offices, boundaries of mining concessions and their characteristics from the Ministry of Energy and Mining (MINEM) and MINAM, and boundaries of logging concessions and their characteristics from MINAG and regional government offices. Further spatially-explicit data layers were obtained from MINAM (roads, rivers, human settlements, forest types and administrative regions) and the Ministry of Transport and Communication (roads). We projected all datasets to WGS 84 UTM zone 18, which were mapped at or resampled (bilinear interpolation) to 30 m resolution.

To account for spatial autocorrelation, and hence model the spatial pattern not explained by other predictor variables^73, 74^, we sampled the data and included the X and Y coordinates of each pixel, their interactions and quadratic terms into the models. This reduced spatial autocorrelation substantially as determined through inspection of Moran’s I correlograms and semi-variograms. Meanwhile, the X and Y coordinates were among the top five predictors of the deforestation and forest degradation models (Supplementary Table S2), suggesting that spatial autocorrelation was high. However, the removal of the X and Y coordinates from the minimal models did not change the direction and form of the relationships between the response and predictor variables. This indicated that the spatial autocorrelation did not significantly alter these relationships, such that further steps to address spatial autocorrelation were not judged necessary.

We drew a stratified random sample of 300,000 pixels from each of the deforestation and forest degradation datasets with a set ratio of 2:1 zeros (no change) to ones (deforested or degraded) in line with Serneels and Lambin^75^. This ratio was chosen as it showed the best goodness-of-fit and normality of residuals, as determined through inspection of Q-Q plots. We checked that the relationships between predictor and response variables were robust when increasing the sample size in the models up to n = 1.2 million, and when varying the ratio of zeros to ones in the response variable.

The minimal adequate model was selected through backward selection from the full model, based on whether the deletion of each term led to an increase in the model’s Akaike’s Information Criterion (AIC)^76^. If the Spearman rank correlation coefficient between two predictor variables was above 0.65^77^, we only maintained the variable with the largest increase in explained deviance in the full model (Supplementary Table S2)^62^. Models goodness-of-fit was evaluated based on their measure of the area under the receiver operating characteristic curve (AUC), exhibiting a very high value for both deforestation (0.93) and forest degradation (0.92).

### Assessing conservation performance

To assess the impacts, if any, of state PAs, CCs and ITs (treatments) on deforestation and forest degradation we used matching, creating artificial control groups for each treatment area while controlling for differences in observed covariates. Based on an assessment of the covariate balance for a variety of matching methods, we chose nearest neighbour matching without replacement using propensity scores and a calliper of 0.25 standard deviations^78^ using the *matchIt* package^79^. We included all treatment PAs (30 state PAs and 13 CCs) in the study area. To make the matching analysis computationally manageable for ITs (n = 992), we randomly selected 439 ITs (44% of 992) of those with at least 50 data pixels. We matched each of the three treatment cohorts to three counterfactual groups: logging concessions, mining concessions and the wider unprotected landscape. To account for potential effects of local leakage^24^ we excluded 1 km (CCs and ITs) and 5 km (state PAs) buffer areas around treatment areas. We also excluded treatment areas established between 2007 and 2011, official state PA buffer areas, inactive logging and mining concessions, areas of overlap, and other types of PAs and forest governance regimes with conservation potential that were region-specific or few in numbers (n < 10) (see Supplementary Methods for details). Given the vast extent of the study area, the treatment and control groups were sampled, drawing a sample of up to 600,000 for each matching analysis as done previously^26^, including up to 100,000 treatment pixels and up to 500,000 control pixels. We randomly sampled up to 10,000 data points within each state PA and CC, and 1,000 data points within each IT because they were more numerous. Because the sample sizes for state PAs and ITs were still above the recommended 100,000 pixels, we ran both of them in three separate sets of 100,000 pixels each.

Covariates used in each matching analysis were selected through an interactive process based on the literature (e.g. refs 24, 26, 28, 30), the results of the GLMs and the observed balance of the covariates. In the initial run of the matching analyses, we included the covariates based on the results of the final GLMs. After each matching run, we assessed the resulting balance of the control and treatment samples across all potential predictor variables (Supplementary Table S1). If there were variables with standardized difference in mean remaining above 0.25^78^, we re-ran the matching analysis including those variables. As the final matching analyses, we chose those that maximised the number of covariates with standardized differences in mean below 0.25. In nearly all cases, matching greatly reduced the mean difference between the control and treatment groups, with eCDF values tending towards zero (Supplementary Tables S7–S15). This provides confidence that differences in deforestation and forest degradation observed between matched treatment and control data points are a treatment effect.

We determined percentage deforestation and degradation rates for each treatment area. We used the paired Wilcoxon test to assess whether there were significant differences in percentage rates of change between treatment areas and the matched controls. Absolute avoided deforestation and degradation was calculated for each treatment area based on the difference between changes observed in matched control and treatment areas. We assessed significant differences, if any, in avoided deforestation and forest degradation between state PAs, CCs and ITs using the unpaired Wilcoxon test. To determine avoided deforestation and degradation relative to rates of change in each of the controls, we divided the absolute avoided deforestation and degradation rates by the deforestation and degradation rates, respectively, in the control area.

### Data Availability

The datasets generated during the current study are available from the corresponding author on reasonable request.

## Electronic supplementary material

Supporting Information
