# Supplementary material for: Conservation performance of different conservation governance regimes in the Peruvian Amazon
Source: Sci Rep. 2017 Sep 12;7:11318. doi: 10.1038/s41598-017-10736-w (PMC5596048; doi:10.1038/s41598-017-10736-w)
Supplement: Supplementary file 1 — Supporting Information [file 41598_2017_10736_MOESM1_ESM.pdf]

Supplementary Information

Conservation performance of different conservation governance regimes in the Peruvian Amazon

Judith Schleicher, Carlos A. Peres, Tatsuya Amano, William Llactayo, Nigel Leader-Williams

Supplementary Methods.

Deforestation and forest degradation analysis

Deforestation and forest degradation were assessed between 2006 and 2011 because (i) a large number of protected areas (PAs), Indigenous Territories (ITs) and Conservation Concessions (CCs) in the Peruvian Amazon were established prior to or in 2006, (ii) Landsat 5 Thematic Mapper images, used for this analysis as providing the best compromise between image resolution, extent and affordability, are only available until the end of 2011, and (iii) this period is particularly interesting as 2005 marked an important turning point concerning the accessibility to the southern Peruvian Amazon with the onset of the pavement of the Madre de Dios (MDD) section of the Inter-Oceanic Highway. The pavement of the road connecting Cuzco with Puerto Maldonado drastically reduced travelling times between the two towns and made the region accessible for heavy mining equipment, dramatically fuelling gold mining activities and associated forest conversion in MDD[1,2].

This study adopted the definitions of forest, deforestation and forest degradation in use at the National Directorate of Land Use Planning (*Dirección General de Ordenamiento Territorial*) in the Peruvian Ministry of Environment (MINAM) and as operationalized through CLASlite. These definitions are specific ones among the multitude of definitions in use for these concepts depending on context[3–5]. In particular, the term forest degradation has been employed to encompass a wide variety of human-induced impacts on forests. In Latin America, this widely includes impacts resulting from selective logging, hunting, logging roads, fire and the extraction of non-timber forest products, such as fuel wood collection[4]. While the analyses conducted here do not seek to incorporate all of these impacts, it does include some of the major ones. Degradation as defined here detects impacts on forests of selective logging, logging roads, secondary regrowth and understory fire.

Accuracy assessment

To assess the accuracy of the deforestation and forest degradation data, we combined field surveys and an assessment using high resolution satellite images. For the fieldwork component, we adapted the protocol developed by Oliveira and colleagues[6] to validate deforestation and forest degradation of a CLASlite predecessor in the Peruvian Amazon. A large field survey was conducted between August and October 2012 in the Pachitea and Ucayali watershed regions (Landsat image path/row 6/66; Supplementary Fig. S2). This region was chosen for the field survey as: (i) it is an area known for high rates of deforestation and selective logging; (ii) it was relatively accessible for field surveys; (iii) we had obtained a nearly

cloud-free image of the area from 2011; and (iv) it is the area where the previous validation survey had taken place[6]. For the field validation, we randomly selected 'forest', 'degraded' and 'deforested' sites from the analysis among those that met the following criteria: (i) affected an area of at least 2.25 ha, calculated based on the root mean square error (RMSE) of the georeferencing accuracy of the Landsat images used in the analysis[7] to ensure that field sites selected for validation would coincide with those on the map generated through the analysis; (ii) located within 1 km of a paved or unpaved road, 500 m within a navigable river, and/or 6 km of the indigenous community Naranjal, south of Turnavista, in order to ensure that the site could be reached within one day walking or driving distance from Pucallpa or Naranjal; and (iii) sites within the same class were at least 1.5 km apart from each other. To maximize the sample size, we visited the field validation sites in clusters, first selecting a 'degraded' site and then a 'forest' and a 'deforested' site within up to 2 km of the 'degraded' site, if possible. A total of 69 field sites were reachable that were classed into 'forest', 'degraded' or 'deforested' by integrating the following information: (i) canopy cover as measured with a concave densitometer, taking four readings at 25 m in each cardinal direction; (ii) signs of human presence (e.g. logged trees, fire marks, walking path, woody debris) within 25 m of the central point; and (iii) a qualitative description of the site's vegetation height and structure, including information about past vegetation changes collected from local people when possible (see Supplementary Table S5).

For the validation with high-resolution satellite images, we used 90 RapidEye images (5 m resolution, 25 by 25 km) from 2011 from across the study area (Supplementary Fig. S2), made available by MINAM. The 90 images were selected based on being nearly cloud free and being taken in the same year as the corresponding Landsat image used in the analysis. We selected a stratified random sample of 588 pixels with at least 100 pixels detected as deforested, following the equation of Tortora[8] and recommendation of Olofsson et al.[9].

The accuracy assessment yielded a 98.1% overall accuracy based on the number of sample plots in the high-resolution satellite images (n=588) and an 85.5% overall accuracy based on the sample plots evaluated during the field survey (n=69), further details of which are given in Supplementary Table S6. Unsurprisingly, the accuracy was therefore lower in areas relatively easily accessible to humans (Supplementary Table S6c) than that of a stratified random sample taken across the study area (Supplementary Table S6a). The user's accuracy for both deforestation and forest degradation was at least 90.8%, while the producer's accuracy was 85.2% or above, based on the number of sample plots. Given that the highest proportion of the study area remains covered in forest, the overall accuracy based on the extent of each class was higher (99.8%; Supplementary Table S6b) than that based on the number of sample plots (Supplementary Table S6a).

It was not possible to separately validate the 2006 forest cover map due to the lack of availability of high resolution satellite image from the required time period. Given that the same methods were applied as for the validated maps and previous assessments, we are confident that the 2006 forest cover map has a similar level of accuracy.

Matching analysis

Matching allows for a counterfactual approach to assess treatment effects, in this case national state PAs, CCs or ITs[10,11]. Through matching, deforestation and degradation rates inside treatment areas can therefore be compared to the rates inside artificial control groups matched according to socio-economic and biophysical factors likely to affect both the location bias of the treatment areas, and deforestation or forest degradation rates[11–13]. We matched the treatment areas to three types of controls, namely logging concessions, mining concessions and the wider unprotected landscape beyond the main official land use designations and mainly under the jurisdiction of the state.

We matched with a calliper of 0.25 standard deviations of the propensity score[14]. If no matching control pixel could be found within this caliper, the treatment pixel was excluded and treatment areas with less than 50 successfully matched pixels were excluded from the analysis. As a result, sample sizes varied between analyses for state PAs and ITs (see Supplementary Table S3), but not CCs (n=13). The order of finding matches (i.e. random, smallest to largest, or largest to smallest propensity score)[15] varied between the individual matching runs, depending on which yielded the best balance.

The size of the buffer areas excluded from the analysis was set to 5 km around state PAs, and 1 km around CCs and ITs as these areas are on average much smaller than state PAs. These buffer sizes were judged meaningful in the national context. We also excluded the official buffer areas, designated by the National Service of Natural Areas Protected by the State (SERNANP) around most of the national state PAs. From the unprotected landscape, we excluded state PAs and CCs designated between 2007 and 2012. We further excluded other types of conservation governance regimes that are region-specific, such as Brazil nut concessions, or those that have small sample sizes such as Indigenous Reserves, regional PAs, and Private Conservation Areas. For mining and logging concessions, we only included those areas that were considered as active during the study period (Supplementary Table S1). We further excluded any areas of overlap between mining and logging concessions, and between mining or logging concessions and the treatment areas. In cases, where there were overlaps between treatment categories, we assigned them to the land use category with the stricter resource use restrictions. It was not possible to account for the presence of agricultural land titles, as these have not been mapped across the country. We could also not include hydrocarbon concessions as (i) they occupy a large proportion of the national territory, leaving few potential areas for matched controls and (ii) the location of the considerably smaller areas where exploration and exploitation activities take place are not disclosed.

Prior to the matching analysis, we performed a power analysis to determine whether the sample size of CCs (n=13) and national state PAs (n=30) would be large enough to detect any potential effects. The power analysis was based on data published in Vuohelainen et al. [16] using GPower 3.1 and confirmed that the sample sizes were sufficiently large (n≥11) to detect an effect size of at least 0.98 at a 0.05 significance level and a power of 0.8.

References

1. Swenson, J. J., Carter, C. E., Domec, J. & Delgado, C. I. Gold Mining in the Peruvian Amazon: Global Prices, Deforestation, and Mercury Imports. *PLoS One* **6,** e18875 (2011).
2. Asner, G. P., Llactayo, W., Tupayachi, R. & Luna, E. R. Elevated rates of gold mining in the Amazon revealed through high-resolution monitoring. *Proc. Natl. Acad. Sci. U. S. A.* **110,** 18454–9 (2013).
3. Sasaki, N. & Putz, F. E. Critical need for new definitions of 'forest' and 'forest degradation' in global climate change agreements. *Conserv. Lett.* **2,** 226–232 (2009).
4. Simula, M. *Towards defining forest degradation: comparative analysis of existing definitions. Forest Resources Assessment Programme working Paper* (2009). at <http://www.ardot.fi/Documents_2/Degradationdefintions.pdf>
5. Thompson, I. D. *et al.* An Operational Framework for Defining and Monitoring Forest Degradation. *Ecol. Soc.* **18,** 20 (2013).
6. Oliveira, P. J. C. *et al.* Land-use allocation protects the Peruvian Amazon. *Science* **317,** 1233–6 (2007).
7. Townshend, J. R. G. *Terrain analysis and Remote Sensing*. (Alten & Unwiss Ltd, 1981).
8. Tortora, R. D. A Note on Sample Size Estimation for Multinomial Populations. *Am. Stat.* **32,** 100–102 (1978).
9. Olofsson, P. *et al.* Good practices for estimating area and assessing accuracy of land change. *Remote Sens. Environ.* **148,** 42–57 (2014).
10. Linkie, M. *et al.* Evaluating biodiversity conservation around a large Sumatran protected area. *Conserv. Biol.* **22,** 683–90 (2008).
11. Andam, K. S., Ferraro, P. J., Pfaff, A., Sanchez-Azofeifa, G. A. & Robalino, J. a. Measuring the effectiveness of protected area networks in reducing deforestation. *Proc. Natl. Acad. Sci. U. S. A.* **105,** 16089–94 (2008).
12. Rosenbaum, P. R. & Rubin, D. B. Constructing a Control Group Using Multivariate Matched Sampling Methods That Incorporate the Propensity Score. *The American Statistician* **39,** 33–38 (1985).
13. Joppa, L. & Pfaff, A. Reassessing the forest impacts of protection: the challenge of nonrandom location and a corrective method. *Ann. N. Y. Acad. Sci.* **1185,** 135–49 (2010).
14. Stuart, E. A. Matching methods for causal inference: A review and a look forward. *Stat. Sci.* **25,** 1–21 (2010).
15. Lunt, M. Selecting an appropriate caliper can be essential for achieving good balance with propensity score matching. *Am. J. Epidemiol.* **179,** 226–235 (2014).
16. Vuohelainen, A. J., Coad, L., Marthews, T. R., Malhi, Y. & Killeen, T. J. The effectiveness of contrasting protected areas in preventing deforestation in Madre de Dios, Peru. *Environ. Manage.* **50,** 645–663 (2012).
17. Green, J. M. H. *et al.* Deforestation in an African biodiversity hotspot: Extent, variation and the effectiveness of protected areas. *Biol. Conserv.* **164,** 62–72 (2013).
18. Nelson, A. *Estimated travel time to the nearest city of 50,000 or more people in year 2000*. (2008). at <http://bioval.jrc.ec.europa.eu/products/gam/index.htm>
19. Jarvis, A., Reuter, H. I., Nelson, A. & Guevara, E. *Hole-filled SRTM for the globe Version 4*. (2008).
20. Sombroek, W. Spatial and Temporal Patterns of Amazon Rainfall. *J. Hum. Environ.* **30,** 388–396 (2001).
21. Hijmans, R. J., Cameron, S. E., Parra, J. L., Jones, P. G. & Javis, A. Very high resolution interpolated climate surfaces for global land areas. *Int. J. Climatol.* **25,** 1965–1978 (2005).

Supplementary Figures and Tables.

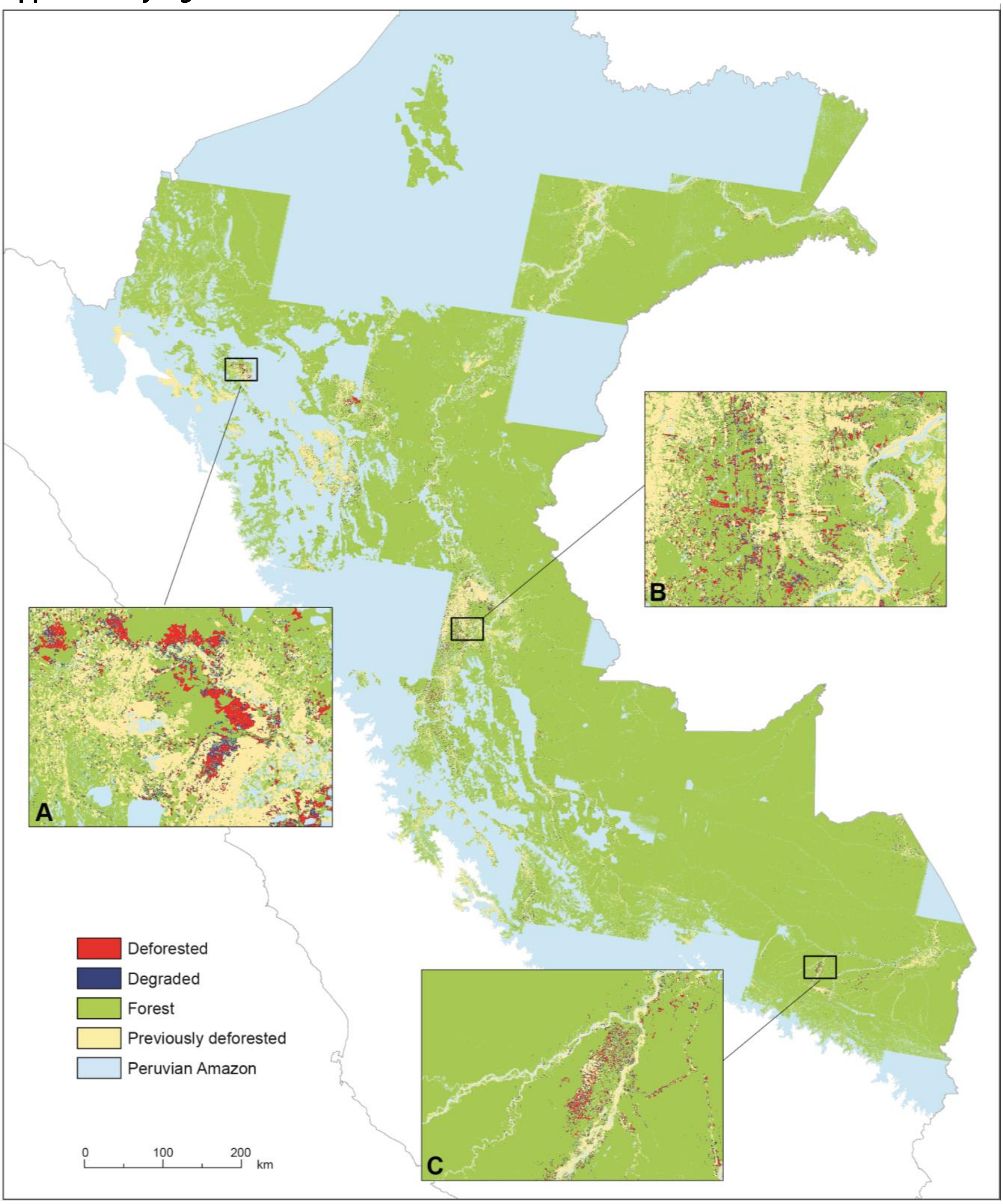

Supplementary Fig. S1. Deforestation and forest degradation between 2006 and 2011 across the study area. Three examples of hotspots of change: A: part of the corridor of agricultural expansion in San Martin (contributing 25% of deforestation and 21% of forest degradation); B: Ucayali's logging center around the city of Pucallpa (41% of deforestation and 35% of degradation); C: mining area south of the city of Puerto Maldonado in eastern Madre de Dios (12% of deforestation and degradation). Source: map produced in Adobe Illustrator CS 5.0 (http://www.adobe.com/uk/products/illustrator.html) based on the analysis of forest change carried out in this study.

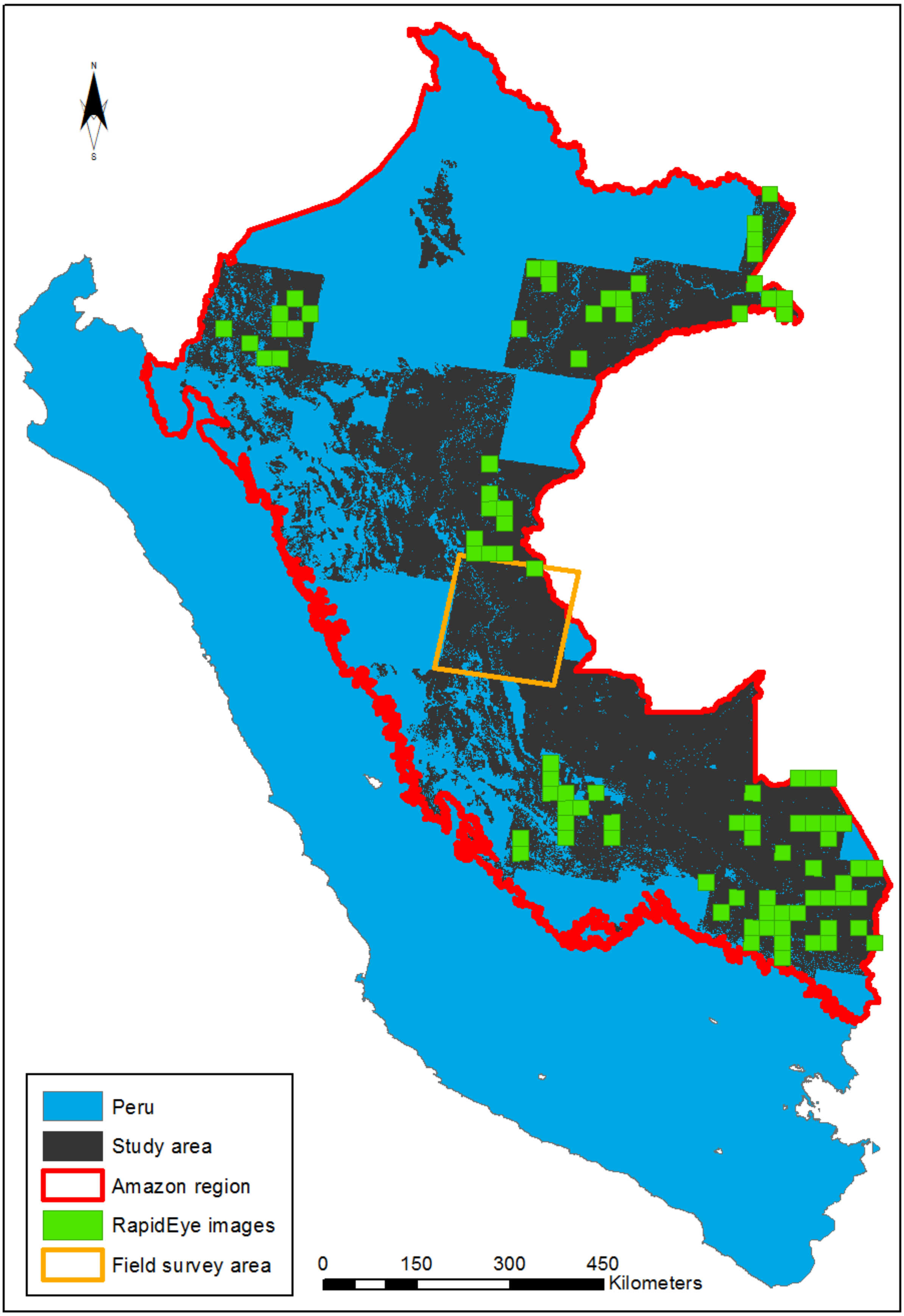

Supplementary Fig. S2. Map of study area of the remote sensing analysis with the location of the RapidEye images used and field survey area for the validation. The map was produced in ArcMap 10.0 (http://desktop.arcgis.com/en/arcmap/) based on data generated and collated for this study.

Supplementary Table S1. Predictor variables included in the analyses, mapped or resampled (bilinear interpolation) at 30m resolution.

**Predictor variable**	**Description**	**Exclusion from models**	**Source**
Distance to previous deforestation (km)	Euclidean distance to the nearest non-forest pixel in 2006 at 30m resolution based on the 2006 forest map generated through processing of Landsat 5 images. This variable was included as deforestation and degradation are expected higher near previous deforestation [17].	-	Own analysis
Distance (km) to:<br>(i) Main roads,<br>(ii) Main & vicinal roads	Euclidean distance to (i) main (national and departmental) roads and (ii) main and vicinal roads. Road layers were obtained from MINAM (2011) and MTC* (2012). Compared layers to maps from previous years and information found online to exclude roads built after 2006, where known.	Distances (i) and (ii) were correlated (>0.65); (i) was included as it explained a larger proportion of the residual deviance.	MTC and MINAM
Distance to rivers (km)	Euclidean distance to main navigable rivers. Layers obtained from MINAM and updated manually by digitizing them in ArcMap 10 to match the rivers in the Landsat images used in the analysis.	-	MINAM
Travel time to markets (h)	Estimated travel time to the nearest city of at least 50,000 people in 2000, based on population centres, transportation networks, topography, land cover and political boundaries. The accessibility map was provided at 30 arc-seconds resolution and was resampled to 30m.	-	[18]
Distance to settlements with at least: (i) 10;<br>(ii) 1,000; (iii) 5,000; and<br>(iv) 10,000 people.	Euclidean distance to settlements of different sizes was calculated based on the 2007 human population data obtained from MINAM. This includes number of inhabitants for all population centres in Peru based on the 2007 national population census.	Distances (i) and (ii) as well as (iii) and (iv) were correlated (>0.65). (i) and (iii) were included in the deforestation model and (i) and (iv) in the degradation model ¶.	MINAM
Population density within:<br>(i) 6 km^2;<br>(ii) 9 km^2; and<br>(iii) 12 km^2	Human population densities in 2007 were estimated in ArcMap 10 from the population data obtained from MINAM (see row above), which is based on the 2007 national population census. Given the larger number of zeros in the resulting data layers (62 to 79%), data were transformed into a binomial variable (presence/absence of settlements) to be modelled adequately.	Distances (i), (ii), and (iii) were correlated (>0.65) with distance to settlements, the latter was retained in the models ¶.	MINAM
Elevation (m)	Elevation was based on the Shuttle Radar Topographic Mission (SRTM) 90m digital elevation data, processed by Jarvis and colleagues [19] for missing data. Layer was resampled to 30m.	Elevation was correlated (>0.65) with slope. Elevation was retained in the degradation model and slope in the deforestation model ¶.	[19]
Slope (°)	Determined the slope using ArcMap's Slope tool, based on the SRTM 90m digital elevation data processed by Jarvis and colleagues [19] for missing data. Layer was resampled to 30m.	See 'elevation'.	[19]
Number of wet months	Calculated number of wet months per year, with >100mm monthly rainfall following [20], using ArcMap's Raster Calculator tool, based on the WorldClim Global Climate data (~1950-2000) provided at 30 arc-seconds resolution and resampled to 30m resolution.	This variables was correlated (>0.65) with rainfall. Number of wet months was retained in the degradation model and rainfall in the deforestation model ¶.	[21]
Rainfall (mm)	Mean annual precipitation data were obtained from the WorldClim Global Climate data (~1950-2000) provided at 30 arc-seconds resolution and resampled to 30m resolution.	See 'number of wet months'.	[21]
Ecoregion	Ecoregions included were *Amazonico*, *Puna* and *Yungas*; excluded *Seco Ecuatorial* as only a small number of data points fell within it. Included *Sabana de palmeras* within *Amazonico* as it could not be modelled independently due to its small size and being restricted to MDD.	-	MINAM
Administrative region	The Peruvian Amazon comprises 14 administrative regions, some of which cover only a small part of the study area and were therefore grouped together into a total of 7 regions.	-	MINAM
Land use designations:<br>(i) National state PAs;<br>(ii) CCs;<br>(iii) Indigenous Territories;<br>(iv) mining concessions;<br>(v) logging concessions.	National PAs' shapefiles were obtained from SERNANP (2012), MINAM (2011) and WWF (2005) to determine which PAs were in place between 2006 and 2011. IT shapefiles were obtained from IBC†. For CCs and logging concessions shapefiles were obtained from MINAG‡ and regional governments. The analysis included only those logging concessions which were active between 2006 and 2011. For mining concessions shapefiles for 2009 and 2011 were obtained from MINEM§ in PSAD 56, as no data prior to 2009 were available. The shapefiles were reprojected to WGS 84 using transformation 8. Only those concessions that were active were included in the analysis, as defined by being granted by 2007 and active in 2011 according to MINAM data.	CCs and mining concessions were not included in the full models because of their relative small extents (comprising only about 1% or less of the sample data points), inhibiting modelling them adequately.	SERNANP, MINAM, WWF, IBC, MINAG regional governments, MINEM
X and Y coordinates	Included the X and Y coordinates (m) as calculated in ArcMap, their interaction and their squared values to account for spatial autocorrelation in the dataset.	-	Own analysis

* MTC: Ministry of Transport and Communication; † IBC: Instituto del Bien Común; ‡ Ministry of Agriculture; § MINEM: Ministry of Energy and Mining; ¶ Variables that explained a larger proportion of the deviance in the null model were included.

Supplementary Table S2. Predictor variables of rates of deforestation and forest degradation. The contribution of the predictor variables to the minimal model was assessed by dropping each predictor variable from the minimal model in turn and calculating the resulting change in the percentage of deviance explained (ΔD) and the change in the Alkaike's Information score (ΔAIC).

	**Deforestation Model**				**Forest Degradation Model**			
**Predictor**	**Estimator**	**S.E.**	**ΔD**	**ΔAIC**	**Estimator**	**S.E.**	**ΔD**	**ΔAIC**
Intercept	-4.762	10.35			-1.50×10^{-3}***	29.52		
Dist. to roads (km)	-5.76×10^{-3}***	2.05×10^{-4}	0.21	796	-6.98×10^{-3}***	2.62×10^{-4}	0.19	718
Dist. to settlements (km)	-0.10***	1.47×10^{-3}	1.71	6547	-0.04***	1.03×10^{-3}	0.46	1765
Dist. to small towns (km)	-5.59×10^{-3}***	2.06×10^{-4}	0.20	756				
Dist. to large towns (km)					-5.17×10^{-6}***	1.54×10^{-7}	0.30	1167
Dist. to previous def.(km)	-1.11***	0.01	3.36	12812	-0.94***	0.01	3.49	13323
Dist. to rivers (km)	0.04***	5.33×10^{-4}	0.18	670				
Travel time to cities (h)	-0.03***	5.75×10^{-4}	0.55	2105	-0.02***	5.03×10^{-4}	0.60	2317
Administrative Regions			1.28	4867			0.41	1591
Loreto	-0.36***	0.04			0.60***	0.04		
MDD	-0.36***	5.99×10^{-3}			0.10***	0.05		
Pasco	-1.49***	0.03			-0.73***	0.03		
San Martin	0.16***	0.04			0.70***	0.04		
Ucayali	-0.84***	0.03			-0.02***	0.03		
Other regions	-1.54***	0.04			-0.11***	0.04		
Slope (°)	-0.08***	1.49×10^{-3}	0.79	3004				
Height (m)					-8.43×10^{-4}***	2.66×10^{-5}	0.28	1078
No. wet months					0.05***	4.69×10^{-4}	0.03	108
Rainfall	4.48×10^{-4}***	1.51×10^{-5}	0.23	877				
Ecoregion: Amazon			0.02	75			0.05	184
Puna	-0.16	0.08			0.62***	0.10		
Yungas	-0.21***	0.02			0.31***	0.02		
StatePAs (ANP)	-1.40***	0.03	0.85	3255	-1.27***	0.02	0.78	2977
Indigenous Comm.	-0.75***	0.02	0.61	2337	-0.65***	0.02	0.44	1668
Logging Concessions	-0.48***	0.02	0.13	513	-0.26***	0.02	0.05	179
X coordinate	-1.24×10^{-4}***	2.01×10^{-6}	1.04	3985	1.25×10^{-5}***	5.12×10^{-6}	<0.01	4
Y coordinate	1.33×10^{-5}***	2.94×10^{-6}	0.01	18	3.38×10^{-4}***	6.29×10^{-6}	1.45	5550
X:Y coordinate	1.20×10^{-11}***	2.97×10^{-13}	1.02	3887	-1.65×10^{-12}***	5.23×10^{-13}	<0.01	8
X coordinate^2	9.78×10^{-12}***	2.11×10^{-13}	0.58	2200	9.19×10^{-13}***	3.54×10^{-13}	<0.01	5
Y coordinate^2	-1.12×10^{-12}***	1.61×10^{-13}	0.02	65	-1.90×10^{-11}***	3.46×10^{-13}	1.63	6242

Significance levels: * significant at $p<0.05$, ** significant at $p<0.01$, *** significant at $p<0.001$.

Note: The pair of predictor variables distance to small and large town, slope and elevation, and rainfall and number of wet months were highly intercorrelated (>0.65). Therefore for each pair only the predictor explaining more of the deviance of the null model was retained in the model. Distance to rivers was dropped from the degradation model as it was not significant.

Supplementary Table S3. Wilcoxon test results of propensity score matching analyses: (A) paired Wilcoxon tests between treatment and matched control areas; and (B) unpaired Wilcoxon tests comparing matching results between different treatments. PA: protected areas; CC: Conservation Concessions; IT: Indigenous Territories.

**(A)**	**State PA**	**CC**	**IT**
**UNPROTECTED MATRIX**			
Deforestation	V=6, n=30, p<0.001	V=12, n=13, p=0.038	V=11323, n=434, p<0.001
Degradation	V=27, n=30, p<0.001	V=14, n=13, p=0.030	V=17322, n=433, p<0.001
**LOGGING CONCESSIONS**			
Deforestation	V=11, n=18, p=0.018	ns	V=4958, n=210, p<0.001
Degradation	V=10, n=18, p=0.005	ns	V=5269, n=207, p<0.001
**MINING CONCESSIONS**			
Deforestation	V=8, n=24, p=0.001	ns	ns
Degradation	V=22, n=24, p=0.001	ns	ns

**(B)**	**CC vs state PA**	**CC vs IT**	**State PA vs IT**
**UNPROTECTED MATRIX**			
Deforestation	W=105, n=13 & 30, p=0.017	ns	W=3529, n=30 & 434, p<0.001
Degradation	W=74, n=13 & 30, p=0.001	ns	W=4032, n=30 & 433, p<0.001
**LOGGING CONCESSIONS**			
Deforestation	ns	ns	ns
Degradation	ns	ns	ns
**MINING CONCESSIONS**			
Deforestation	ns	ns	ns
Degradation	ns	ns	W=1780, n=24 & 115, p=0.025

Supplementary Table S4. Details of the Landsat 5 scenes included in the analysis

Path/Row	#	Date Year 1		#	Date Year 2
2/69	1	31 July 2007		1	10 July 2011
3/68	2	4 Aug 2006		2	30 July 2010*
				3	18 Aug 2011*
3/69	3	17 June 2006		4	3 Sept 2011**
4/62	4	29 July 2007		5	9 Aug 2011
4/63	5	24 June 2006		6	9 Aug 2011**
4/67	6	24 June 2006*		7	25 Aug 2011
	7	28 Sept 2006*			
4/68	8	26 May 2007		8	6 Aug 2011
4/69	9	11 June 2007*		9	6 Aug 2010*
	10	28 Sept 2006*		10	6 June 2011*
5/63	11	17 July 2006		11	14 Sept 2010
5/67	12	14 May 2006		12	28 July 2010
5/68	13	30 July 2005		13	16 Aug 2011*[&]**
				14	13 June 2011*[&]**
6/63	14	19 June 2005*		15	20 June 2011*
	15	6 Aug 2005*		16	7 Aug 2011*
6/65	16	5 July 2005*		17	7 Aug 2011**
	17	19 June 2005*			
	18	9 Aug 2006*			
6/66	19	5 May 2006*		18	7 Aug 2011**
	20	22 Aug 2005*			
	21	5 July 2005*			
	22	14 Aug 2008*			
6/67	23	22 Apr 2007*		19	22 July 2011*
	24	5 May 2006*		20	7 Aug 2011*
	25	21 July 2005*		21	19 July 2010*
	26	26 Sept 2006*			
6/68	27	9 Aug 2006*		22	17 June 2010*
	28	19 June 2005*		23	16 May 2010*
	29	9 June 2007*		24	7 Aug 2011*
7/62	30	12 May 2006*		25	15 Sept 2011*
	31	8 Feb 2007*		26	2 Jan 2011*
7/64	32	12 May 2006*		27	15 Sept 2011
	33	12 July 2005*			
7/65	34	2 July 2007*		28	29 July 2011*
	35	18 July 2007*		29	30 Aug 2011*
	36	28 May 2006*		30	15 Sept 2011*
	37	1 Sept 2006*		31	28 Sept 2010*
				32	1 Oct 2011*
7/67	38	28 July 2005		33	11 Aug 2010*
				34	14 Aug 2011*
8/62	39	8 Sept 2006		35	6 Sept 2011
8/64	40	10 Aug 2007*		36	20 July 2011*
	41	17 June 2005*		37	18 Aug 2010*
	42	30 Jan 2007*			
	43	8 Sept 2006*			
8/65	44	22 July 2006*		38	14 May 2010*
	45	8 Sept 2006*		39	18 Aug 2010*
				40	20 July 2011*
				41	15 June 2010*
9/63	46	1 Aug 2007		42	12 Aug 2011
9/64	47	15 Sept 2006*		43	12 Aug 2011*
	48	1 Aug 2007*		44	14 Feb 2010*

* Combined the images to minimize cloud cover, cut in ENVI using the ROI tool; ** To optimize cloud and water masking, cut and combined images of different levels of masking.

Supplementary Table S5. Field validation results (VAL) of field plots (n=69) compared to the results of the remote sensing analysis (Analysis).

#	Analysis	Qualitative assessment	H*	Canopy cover †: N	S	E	W	ALL	VAL
1	DEG	no forest cover, recent fire, some trees	yes	DEF	DEF	DEF	DEF	DEF	DEF
2	DEF	no tree cover, burned	yes	DEF	DEF	DEF	DEF	DEF	DEF
3	DEF	pasture, some palm trees	yes	DEF	DEF	DEF	DEF	DEF	DEF
4	DEG	pasture, dry trees, logged trees	yes	DEF	DEF	DEF	DEF	DEF	DEF
5	F	disturbed mature forest	no	F	F	F	F	F	F
6	F	mature forest, fallen trees	no	F	F	F	F	F	F
7	DEG	secondary regrowth, some trees	yes	F	DEF	DEF	IMP	DEF	DEF
8	DEF	agriculture	yes	DEF	DEF	DEF	DEF	DEF	DEF
9	DEG	dense secondary regrowth	yes	F	F	F	F	F	DEG
10	DEF	secondary regrowth, burned	yes	DEF	DEF	DEF	DEF	DEF	DEF
11	DEG	secondary regrowth, recent fire	yes	DEF	DEF	DEF	DEF	DEF	DEF
12	F	highly disturbed mature forest	no	DEF	F	F	F	F	NC
13	DEF	logged forest, secondary regrowth	yes	DEF	DEF	IMP	DEF	DEF	DEF
14	DEG	secondary regrowth, trees, part agriculture	yes	IMP	DEF	DEF	DEF	DEF	DEF
15	DEG	recent fire, dry trees, secondary regrowth	yes	IMP	DEF	DEF	DEF	DEF	DEF
16	F	disturbed mature forest	no	F	F	F	F	F	F
17	DEF	secondary regrowth, next to agriculture	yes	IMP	F	DEF	DEF	DEF	DEF
18	DEF	dry/fallen trees, recent fire	yes	DEF	DEF	DEF	DEF	DEF	DEF
19	DEG	secondary regrowth, fallen trees, logged trees	yes	DEF	DEF	DEF	DEF	DEF	DEF
20	F	very recent logging, previously mature forest	yes	DEF	F	IMP	IMP	IMP	F
21	DEG	secondary regrowth, fallen trees, some trees	no	IMP	F	F	F	F	F
22	DEF	agriculture, burned, palm trees, dry trees	yes	DEF	DEF	DEF	DEF	DEF	DEF
23	F	highly disturbed forest	yes	DEF	IMP	DEF	DEF	DEF	DEF
24	DEG	highly disturbed forest, fallen trees	no	F	DEF	F	IMP	IMP	DEG
25	DEF	disturbed mature forest, agriculture	yes	DEF	F	DEF	F	DEF	DEF
26	DEG	highly disturbed forest, fallen trees	no	IMP	F	F	IMP	IMP	DEG
27	F	mature forest (aguajal)	no	F	F	F	F	F	F
28	F	disturbed mature forest	no	F	F	F	F	F	F
29	F	secondary regrowth, fallen/dry trees	no	IMP	F	DEF	DEF	DEF	DEF
30	DEG	logged, secondary regrowth, palm trees, burned	yes	DEF	DEF	DEF	DEF	DEF	DEF
31	DEF	logged forest, burned, dry trees, regrowth	yes	DEF	DEF	DEF	DEF	DEF	DEF
32	DEG	secondary regrowth, disturbed forest, palm trees	yes	DEF	F	DEF	F	DEF	DEF
33	F	secondary regrowth, burned	yes	F	DEF	DEF	IMP	DEF	DEF
34	DEF	secondary regrowth, dry trees, burned	yes	DEF	DEF	DEF	DEF	DEF	DEF
35	DEF	secondary regrowth, burned	yes	DEF	DEF	DEF	DEF	DEF	DEF

#	Analysis	Qualitative assessment	H	Canopy cover: N	S	E	W	ALL	VAL
36	DEG	secondary regrowth, fallen/logged trees	yes	DEF	DEF	DEF	DEF	DEF	DEF
37	DEG	secondary regrowth, recent fire	yes	DEF	DEF	DEF	IMP	DEF	DEF
38	DEF	abandoned fields, burned, secondary regrowth	yes	DEF	DEF	DEF	DEF	DEF	DEF
39	DEG	secondary regrowth, some palm trees	yes	DEF	DEF	DEF	F	DEF	DEF
40	F	mature forest (renaqual)	no	F	DEF	F	DEF	DEF	NC
41	DEG	secondary regrowth, burned, logged & fallen trees	yes	DEF	IMP	IMP	IMP	IMP	DEG
42	F	palm oil plantation	yes	DEF	IMP	F	F	IMP	DEF
43	DEF	secondary regrowth, palm oil plantation	yes	DEF	IMP	DEF	DEF	DEF	DEF
44	DEF	palm oil plantation	yes	F	IMP	DEF	F	IMP	DEF
45	DEG	secondary regrowth, dry trees	yes	DEF	IMP	DEF	DEF	DEF	DEF
46	DEF	secondary regrowth, logged forest	yes	DEF	F	DEF	F	DEF	DEF
47	DEG	secondary regrowth, disturbed mature forest	yes	F	F	F	F	F	DEG
48	F	secondary regrowth, disturbed forest, palm trees	yes	F	IMP	F	IMP	IMP	DEG
49	DEF	agriculture, recent fire	yes	F	DEF	DEF	DEF	DEF	DEF
50	DEF	disturbed forest	no	F	F	F	F	F	F
51	DEG	disturbed forest, fallen trees, secondary regrowth	no	F	F	F	IMP	F	F
52	F	mature forest, fallen tree	yes	F	F	F	F	F	DEG
53	DEG	secondary regrowth	no	IMP	DEF	IMP	IMP	IMP	DEG
54	DEF	secondary regrowth	yes	F	F	F	F	F	DEG
55	F	natural secondary regrowth	no	F	F	F	F	F	NC
56	F	mature forest, fallen trees	no	F	F	F	F	F	F
57	DEF	agriculture	yes	DEF	DEF	DEF	DEF	DEF	DEF
58	DEG	secondary regrowth, dry/fallen trees, palm trees	yes	IMP	IMP	DEF	IMP	IMP	DEG
59	DEF	pasture, some palm trees, some dry trees	yes	DEF	DEF	DEF	DEF	DEF	DEF
60	DEG	pasture, some palm trees, dry/fallen tree, burned	yes	DEF	DEF	DEF	DEF	DEF	DEF
61	DEG	secondary regrowth, some trees, some pasture	yes	DEF	DEF	IMP	IMP	DEF	DEF
62	DEF	secondary regrowth, burned	yes	DEF	DEF	DEF	DEF	DEF	DEF
63	DEG	forest plantation	yes	IMP	F	DEF	F	IMP	DEG
64	F	mature forest	no	F	F	F	F	F	F
65	DEF	logged forest, burned, secondary regrowth	yes	DEF	DEF	DEF	DEF	DEF	DEF
66	DEF	logged forest, burned, secondary regrowth	yes	DEF	DEF	DEF	DEF	DEF	DEF
67	DEG	very recently logged, recent fire	yes	DEF	DEF	DEF	DEF	DEF	DEF
68	DEF	pasture, logged forest, burned, secondary regrowth	yes	DEF	DEF	DEF	DEF	DEF	DEF
69	F	mature forest	no	F	F	F	F	F	F

DEF: deforested; F: forest; DEG: degraded; IMP: impacted; NC: natural change. * H = Human presence (yes/no); † Canopy cover as assessed by densiometer in four sub-plots (North, N; South, S; East, E; West, W), integrated into an overall assessment (ALL), according to the following criteria: (1) DEF: if ≥ 2 sub-plots DEF; otherwise (2) IMP: if ≥ 2 sub-plots IMP, or 1 IMP and 1 DEF; (3) F: if ≥ 3 sub-plots F.

Supplementary Table S6. Accuracy Assessment of deforestation and forest degradation analysis, based on high resolution RapidEye satellite images (A-B) and based on a fieldwork survey (C). (A) and (C) show accuracy values based on the number of sample plots per class, while (B) shows accuracy in terms of the area of each class.

**(A)**	**CLASlite analysis:**			
**RapidEye Validation:**	**Forest**	**Deforested**	Total	Producer's Accuracy (%)
**Forest**	478	10	488	97.95
**Deforested**	1	99	100	99.00
Total	479	109	588	**Overall:**
User's Accuracy (%)	99.79	90.83		98.13

**(B)**	**CLASlite Analysis:**			
**RapidEye Validation:**	**Forest**	**Deforested**	Total	Producer's Accuracy (%)
**Forest**	0.994	<0.001	0.995	99.97
**Deforested**	0.002	0.003	0.005	60.83
Total	0.996	0.004	1.000	**Overall:**
User's Accuracy (%)	99.79	90.83		99.76

**(C)**	**CLASlite analysis:**				
**Field survey:**	**Forest**	**Degraded**	**Deforested**	Total	Producer's Accuracy (%)
**Forest**	12	2	1	15	80.00
**Degraded**	2	24	1	27	88.89
**Deforested**	4	0	23	27	85.19
Total	18	26	25	69	**Overall:**
User's accuracy (%)	66.67	92.31	92.00		85.51

Supplementary Table S7. Covariate balance before and after matching data points in Conservation Concessions (CC, n=81,001) and the wider unprotected matrix (control, n=500,000). Successfully matched: n=67,304.

		Mean CC	Mean Control	SD Control	Std mean difference	eCDF Med *	eCDF Mean *	eCDF Max *
**PS distance**	Before	0.39	0.09	0.14	1.15	0.43	0.38	0.62
	After	0.33	0.32	0.22	0.03	0.01	0.02	0.06
**Road (km)**	Before	49.50	79.02	78.30	-0.64	0.11	0.12	0.25
	After	54.56	58/38	60.64	-0.08	0.05	0.06	0.17
**Settlement**	Before	9.60	11.74	10.78	-0.27	0.05	0.05	0.12
**(km)**	After	8.78	7.65	6.34	0.14	0.03	0.03	0.06
**Forest edge**	Before	0.91	1.24	1.70	-0.25	0.01	0.02	0.08
**(km)**	After	0.89	0.83	1.21	0.05	0.01	0.01	0.06
**Rivers (km)**	Before	17.66	14.93	13.18	0.19	0.05	0.06	0.14
	After	15.67	14.59	14.48	0.07	0.04	0.06	0.15
**Slope**	Before	7.75	3.33	5.95	0.41	0.12	0.11	0.20
	After	6.52	6.19	9.30	0.03	0.01	0.01	0.03
**Pop.density**	Before	0.17	0.23	0.42	-0.15	0.03	0.03	0.06
**(6 km^2)**	After	0.21	0.24	0.43	-0.09	0.02	0.02	0.04
**Travel time**	Before	24.37	30.65	19.82	-0.52	0.05	0.06	0.17
**(h)**	After	24.08	23.40	16.85	0.05	0.05	0.05	0.16
**Towns (km)**	Before	72.46	79.32	49.26	-0.16	0.04	0.05	0.14
	After	72.82	74.29	51.48	-0.04	0.05	0.05	0.12
**Wet months**	Before	9.53	10.66	1.98	-0.49	0.02	0.09	0.29
	After	9.73	9.81	2.33	-0.04	0.01	0.02	0.11
**Rainfall (mm)**	Before	2077	2349	578	-0.43	0.10	0.13	0.30
	After	2119	2135	573	-0.03	0.03	0.05	0.16
**Large towns**	Before	100.50	120.46	84.50	-0.39	0.07	0.07	0.19
**(km)**	After	101.34	100.62	69.65	0.01	0.05	0.05	0.14
**Towns (1k)**	Before	31.98	45.55	35.89	-0.77	0.13	0.12	0.21
**(km)**	After	32.12	32.55	23.23	-0.02	0.06	0.07	0.15

* eCDF Med/Mean/Max: Median/Mean/Maximum differences in empirical cumulative distribution functions.

Supplementary Table S8. Covariate balance before and after matching data points in Conservation Concessions (CC, n=81,001) and logging concessions (control, n=500,000). Successfully matched: n=56,058.

		Mean CC	Mean Control	SD Control	Std mean difference	eCDF Med *	eCDF Mean *	eCDF Max *
**PS distance**	Before	0.54	0.07	0.15	1.58	0.46	0.44	0.77
	After	0.42	0.40	0.24	0.05	0.02	0.02	0.05
**Road (km)**	Before	49.50	83.57	54.41	-0.74	0.22	0.23	0.43
	After	55.23	51.57	49.67	0.08	0.04	0.07	0.18
**Settlement**	Before	9.60	20.55	14.11	-1.38	0.24	0.22	0.43
**(km)**	After	10.00	9.32	7.20	0.09	0.04	0.04	0.10
**Forest edge**	Before	0.91	2.23	2.55	-0.10	0.04	0.07	0.31
**(km)**	After	1.04	1.15	1.29	-0.09	0.01	0.02	0.12
**Rivers (km)**	Before	17.66	18.35	12.33	-0.05	0.03	0.04	0.12
	After	14.60	12.87	9.31	0.12	0.05	0.05	0.08
**Slope**	Before	7.75	2.86	2.67	0.46	0.04	0.08	0.24
	After	4.25	3.09	4.40	0.11	0.02	0.02	0.06
**Pop.density**	Before	0.17	0.05	0.22	0.32	0.06	0.06	0.12
**(per 6 km^2)**	After	0.15	0.16	0.37	-0.04	0.01	0.01	0.02
**Travel time**	Before	24.37	42.18	19.26	-1.46	0.17	0.19	0.43
**(h)**	After	24.62	21.69	13.04	0.24	0.02	0.05	0.16
**Towns (km)**	Before	72.46	86.68	34.69	-0.34	0.18	0.17	0.31
	After	74.34	72.06	46.78	0.05	0.06	0.08	0.19
**Wet months**	Before	9.53	9.27	1.90	0.11	0.04	0.06	0.17
	After	9.59	9.58	2.27	0.00	0.01	0.02	0.09
**Rainfall (mm)**	Before	2077	2141	450	-0.10	0.04	0.07	0.33
	After	2131	2195	621	-0.10	0.03	0.05	0.18
**Ecoregion**	Before	0.07	0.00	0.05	0.26	0.03	0.03	0.06
**Puna**	After	0.02	0.02	0.13	0.03	0.00	0.00	0.01
**Ecoregion**	Before	0.23	0.01	0.11	0.52	0.11	0.11	0.22
**Yungas**	After	0.13	0.07	0.25	0.15	0.03	0.03	0.06

* eCDF Med/Mean/Max: Median/Mean/Maximum differences in empirical cumulative distribution functions.

Supplementary Table S9. Covariate balance before and after matching data points in Conservation Concessions (CC, n=81,001) and mining concessions (n=500,000). Successfully matched: n=15,010.

		Mean CC	Mean Control	SD Control	Std mean difference	eCDF Med *	eCDF Mean *	eCDF Max *
**PS distance**	Before	0.83	0.03	0.09	2.88	0.50	0.48	0.89
	After	0.35	0.33	0.31	0.09	0.03	0.06	022
**Road (km)**	Before	49.50	9.01	8.07	0.88	0.52	0.46	0.61
	After	21.12	16.75	18.32	0.09	0.08	0.11	0.29
**Settlement**	Before	9.60	3.61	2.30	0.76	0.27	0.26	0.46
**(km)**	After	4.55	4.56	3.24	0.00	0.02	0.02	0.06
**Forest edge**	Before	0.91	0.50	0.80	0.31	0.02	0.04	0.23
**(km)**	After	0.84	0.72	1.37	0.09	0.06	0.07	0.03
**Rivers (km)**	Before	17.66	8.78	12.73	0.61	0.13	0.18	0.40
	After	14.67	16.72	20.88	-0.14	0.14	0.14	0.30
**Slope**	Before	7.75	7.05	9.25	0.07	0.03	0.03	0.05
	After	12.28	11.77	12.84	0.05	0.03	0.03	0.07
**Pop. density**	Before	0.17	0.56	0.50	-1.04	0.20	0.20	0.40
**(6 km^2)**	After	0.43	0.47	0.50	-0.10	0.03	0.02	0.04
**Pop. density**	Before	0.51	0.90	0.30	-0.78	0.20	0.20	0.40
**(12 km^2)**	After	0.85	0.80	0.40	0.10	0.03	0.03	0.05
**Large towns**	Before	100.52	105.64	443.63	-0.10	0.08	0.10	0.25
**(km)**	After	721.62	84.01	51.31	-0.23	0.08	0.10	0.29
**Wet months**	Before	9.53	11.23	1.75	-0.73	0.02	0.14	0.50
	After	9.34	9.44	3.00	-0.05	0.08	0.07	0.16
**Rainfall (mm)**	Before	2077	3639	1090	-2.50	0.37	0.35	0.66
	After	2316	2590	1207	-0.43	0.09	0.10	0.26
**Ecoregion 1**	Before	0.07	0.02	0.14	0.19	0.02	0.02	0.05
**Puna**	After	0.01	0.01	0.09	0.01	0.00	0.00	0.00
**Ecoregion 2**	Before	0.23	0.17	0.37	0.16	0.03	0.03	0.07
**Yungas**	After	0.43	0.39	0.49	0.08	0.02	0.02	0.03

* eCDF Med/Mean/Max: Median/Mean/Maximum differences in empirical cumulative distribution functions.

Supplementary Table S10. Covariate balance before and after matching data points in state protected areas (PA, n=70,612) and the unprotected matrix (n=500,000). Successfully matched: n=33,905.

		Mean PA	Mean Control	SD Control	Std mean difference	eCDF Med *	eCDF Mean *	eCDF Max *
**PS distance**	Before	0.68	0.05	0.13	2.15	0.49	0.47	0.86
	After	0.46	0.44	0.26	0.06	0.02	0.02	0.03
**Road (km)**	Before	36.62	79.02	78.30	-1.53	0.19	0.17	0.30
	After	41.94	37.00	40.00	0.18	0.08	0.08	0.12
**Settlement**	Before	17.30	11.75	10.80	0.42	0.17	0.14	0.20
**(km)**	After	16.96	16.77	16.51	0.01	0.05	0.05	0.11
**Forest edge**	Before	0.84	1.24	1.70	-0.29	0.01	0.03	0.15
**(km)**	After	1.00	1.00	1.67	0.00	0.01	0.01	0.09
**Rivers (km)**	Before	25.13	14.94	13.17	0.44	0.17	0.15	0.22
	After	23.13	22.64	18.50	0.02	0.05	0.05	0.09
**Slope**	Before	16.16	3.33	5.95	1.13	0.37	0.37	0.67
	After	12.88	13.21	12.64	-0.03	0.07	0.07	0.16
**Pop. density**	Before	0.11	0.23	0.42	-0.37	0.06	0.06	0.12
**(6 km^2)**	After	0.17	0.18	0.38	-0.03	0.00	0.00	0.01
**Travel time (h)**	Before	26.82	30.65	19.81	-0.21	0.02	0.04	0.13
	After	26.42	25.34	17.98	0.06	0.03	0.04	0.09
**Towns (km)**	Before	61.61	79.32	49.26	-0.40	0.13	0.12	0.19
	After	68.41	68.30	36.27	0.00	0.03	0.05	014
**Wet months**	Before	8.31	10.66	1.98	-0.90	0.14	0.18	0.49
	After	8.83	8.77	3.08	0.03	0.05	0.05	0.13
**Rainfall (mm)**	Before	1921	3449	578	-0.58	0.21	0.18	0.36
	After	2094	2094	875	0.00	0.05	0.05	0.09
**Elevation (m)**	Before	1259	267	374	1.12	0.20	0.27	0.78
	After	960	952	881	0.01	0.02	0.03	0.28
**Ecoregion 1**	Before	0.06	0.00	0.07	0.24	0.03	0.03	0.06
**Puna**	After	0.06	0.05	0.22	0.03	0.00	0.00	0.01
**Ecoregion 2**	Before	0.64	0.08	0.27	1.17	0.28	0.28	0.56
**Yungas**	After	0.48	0.50	0.50	-0.4	0.01	0.01	0.02

* eCDF Med/Mean/Max: Median/Mean/Maximum differences in empirical cumulative distribution functions.

Supplementary Table S11. Covariate balance before and after matching data points in state protected areas (PA, n=84,867) and logging concessions (n=500,000). Successfully matched: n=40,802.

		Mean PA	Mean Control	SD Control	Std mean difference	eCDF Med *	eCDF Mean *	eCDF Max *
**PS distance**	Before	0.63	0.06	0.10	1.50	0.47	0.42	0.70
	After	0.28	0.25	0.22	0.08	0.04	0.05	0.13
**Road (km)**	Before	50.83	83.57	54.41	-0.75	0.19	0.20	0.34
	After	72.71	68.26	48.73	0.10	0.06	0.06	011
**Settlement**	Before	16.28	20.55	14.11	-0.32	0.04	0.09	0.22
**(km)**	After	18.94	18.95	14.13	0.00	0.01	0.01	0.04
**Forest edge**	Before	1.43	2.23	2.55	-0.36	0.03	0.04	0.24
**(km)**	After	1.76	1.92	2.07	-0.07	0.01	0.01	0.03
**Rivers (km)**	Before	27.59	18.35	12.33	0.39	0.144	0.13	0.22
	After	17.33	18.04	11.66	-0.03	0.05	0.07	0.15
**Slope**	Before	10.36	2.86	2.67	0.60	0.09	0.12	0.34
	After	2.15	2.28	2.41	-0.01	0.05	0.05	0.09
**Pop. density**	Before	0.16	0.05	0.22	0.31	0.06	0.06	0.11
**(6 km^2)**	After	0.08	0.08	0.27	0.00	0.00	0.00	0.00
**Travel time**	Before	30.31	42.18	19.26	-0.68	0.14	0.13	0.24
**(h)**	After	32.00	33.91	17.32	-0.11	0.02	0.03	0.10
**Towns**	Before	84.45	86.69	34.39	-0.03	0.16	0.16	0.26
**(km)**	After	84.68	80.50	38.98	0.06	0.03	0.04	0.11
**Wet months**	Before	9.40	9.27	1.20	0.05	0.12	0.11	0.21
	After	10.88	10.59	1.80	0.10	0.03	0.04	0.14
**Rainfall (mm)**	Before	2068	2141	450	-0.11	0.11	0.11	0.27
	After	2426	2372	520	0.08	0.08	0.09	0.23
**Elevation (m)**	Before	868	276	147	0.58	0.16	0.16	0.34
	After	204	219	119	-0.02	0.00	0.04	0.20
**Ecoregion 1**	Before	0.03	0.00	0.05	0.17	0.01	0.01	0.03
**Puna**	After	0.00	0.00	0.03	0.00	0.00	0.00	0.00
**Ecoregion 2**	Before	0.34	0.01	0.11	0.69	0.16	0.16	0.33
**Yungas**	After	0.00	0.01	0.01	-0.01	0.00	0.00	0.01

* eCDF Med/Mean/Max: Median/Mean/Maximum differences in empirical cumulative distribution functions.

Supplementary Table S12. Covariate balance before and after matching one cohort of data points in state protected areas (PA, n=85,365) and mining concessions (n=500,000). Successfully matched: n=5,059.

		Mean PA	Mean Control	SD Control	Std mean difference	eCDF Med *	eCDF Mean *	eCDF Max *
**PS distance**	Before	0.95	0.01	0.06	5.92	0.51	0.51	0.97
	After	0.45	0.40	0.33	0.34	0.06	0.07	0.15
**Road (km)**	Before	73.81	9.01	8.07	1.20	0.64	0.56	0.78
	After	33.98	24.02	24.71	0.18	0.13	0.12	0.24
**Settlement**	Before	19.65	3.61	2.30	0.95	0.46	0.44	0.76
**(km)**	After	10.37	7.49	5.43	0.17	0.03	0.05	0.17
**Forest edge**	Before	1.55	0.50	0.80	0.47	0.07	0.09	0.36
**(km)**	After	1.23	0.87	1.71	0.16	0.05	0.07	0.18
**Rivers (km)**	Before	17.13	8.78	12.73	0.72	0.18	0.19	0.48
	After	14.74	13.61	13.51	0.10	0.11	0.12	0.26
**Slope**	Before	12.29	7.04	9.25	0.57	0.23	0.22	0.41
	After	11.14	12.40	12.39	-0.14	0.08	0.07	0.15
**Pop. density**	Before	0.06	0.54	0.50	-2.19	0.25	0.25	0.51
**(6 km^2)**	After	0.29	0.38	0.49	-0.40	0.05	0.05	0.09
**Travel time (h)**	Before	32.05	8.97	4.70	1.44	0.24	0.31	0.75
	After	20.85	18.45	13.35	0.15	0.03	0.06	0.25
**Towns (km)**	Before	133.09	86.31	33.57	0.65	0.06	0.10	0.45
	After	92.69	82.13	41.22	0.15	0.04	0.05	0.17
**Wet months**	Before	9.18	11.23	1.75	-1.03	0.03	0.18	0.57
	After	9.88	10.31	2.62	-0.22	0.06	0.09	0.25
**Pop. density**	Before	0.10	0.80	0.40	-2.31	0.35	0.35	0.70
**(9 km^2)**	After	0.41	0.047	0.50	-0.21	0.03	0.03	0.06
**Pop. density**	Before	0.15	0.90	0.30	-2.06	0.37	0.37	0.75
**(12 km^2)**	After	0.51	0.56	0.50	-0.16	0.03	0.03	0.06
**Ecoregion 1**	Before	0.00	0.02	0.14	-0.70	0.01	0.01	0.02
**Puna**	After	0.01	0.01	0.08	0.09	0.00	0.00	0.00
**Ecoregion 2**	Before	0.55	0.17	0.37	0.76	0.19	0.19	0.38
**Yungas**	After	0.41	0.45	0.50	-0.09	0.02	0.02	0.05

* eCDF Med/Mean/Max: Median/Mean/Maximum differences in empirical cumulative distribution functions.

Supplementary Table S13. Covariate balance before and after matching one cohort of data points in Indigenous Territories (IT, n=95,713) and in the wider unprotected matrix (n=500,000). Successfully matched: n=79,191.

		Mean IT	Mean Control	SD Control	Std mean difference	eCDF Med *	eCDF Mean *	eCDF Max *
**PS distance**	Before	0.42	0.11	0.15	1.26	0.42	0.37	0.58
	After	0.35	0.34	0.21	0.04	0.01	0.01	0.03
**Road (km)**	Before	55.29	79.00	78.29	-0.39	0.09	0.10	0.20
	After	59.06	59.48	67.64	-0.01	0.07	0.07	0.12
**Settlement**	Before	4.28	11.75	10.80	-2.22	0.18	0.20	0.40
**(km)**	After	4.55	4.70	4.63	-0.04	0.02	0.02	0.05
**Forest edge**	Before	0.61	1.24	1.70	-0.80	0.03	0.04	0.18
**(km)**	After	0.66	0.64	1.01	0.02	0.01	0.01	0.10
**Rivers (km)**	Before	9.55	14.93	13.18	-0.40	0.12	0.12	0.23
	After	10.07	10.25	12.38	-0.02	0.01	0.01	0.04
**Slope**	Before	5.28	3.33	5.95	0.29	0.10	0.10	0.20
	After	4.96	4.96	7.74	0.00	0.02	0.02	0.05
**Pop. density**	Before	0.48	0.23	0.42	0.51	0.13	0.13	0.25
**(6 km^2)**	After	0.46	0.47	0.50	-0.01	0.00	0.00	0.01
**Travel time (h)**	Before	21.23	30.65	19.81	-0.68	0.06	0.09	0.37
	After	21.94	22.20	18.84	-0.02	0.02	0.04	0.15
**Towns (km)**	Before	88.80	79.31	49.27	0.19	0.07	0.06	0.12
	After	81.70	77.53	53.43	0.08	0.04	0.05	0.10
**Wet months**	Before	9.81	10.66	1.98	-0.43	0.03	0.08	0.27
	After	9.67	9.60	2.04	0.04	0.01	0.02	0.04
**Rainfall (mm)**	Before	2162	2349	578	-0.39	0.10	0.11	0.23
	After	2126	2094	525	0.03	0.01	0.02	0.06
**Ecoregion 1**	Before	0.00	0.00	0.07	-	0.00	0.00	0.00
**Puna**	After	0.00	0.00	0.02	-	0.00	0.00	0.00
**Ecoregion 2**	Before	0.20	0.08	0.27	0.31	0.06	0.06	0.12
**Yungas**	After	0.17	0.16	0.37	0.03	0.01	0.01	0.01

* eCDF Med/Mean/Max: Median/Mean/Maximum differences in empirical cumulative distribution functions.

Supplementary Table S14. Covariate balance before and after matching one cohort of data points in Indigenous Territories (IT, n=95,713) and logging concessions (n=500,000). Successfully matched: n=35,692.

		Mean PA	Mean Control	SD Control	Std mean difference	eCDF Med *	eCDF Mean *	eCDF Max *
**PS distance**	Before	0.75	0.05	0.12	2.35	0.50	0.47	0.83
	After	0.43	0.38	0.23	0.17	0.05	0.06	0.11
**Road (km)**	Before	55.29	83.56	54.41	-0.46	0.24	0.21	0.34
	After	76.78	78.50	61.70	-0.03	0.07	0.07	0.17
**Settlement**	Before	4.28	20.55	14.11	-4.84	0.29	0.32	0.70
**(km)**	After	5.36	6.99	7.53	-0.49	0.06	0.05	0.08
**Forest edge**	Before	0.61	0.22	2.55	-2.06	0.05	0.08	0.41
**(km)**	After	0.88	1.02	1.28	-0.17	0.01	0.01	0.05
**Rivers (km)**	Before	9.56	18.35	12.33	-0.81	0.18	0.19	0.40
	After	10.43	11.30	9.60	-0.08	0.08	0.07	0.14
**Slope**	Before	5.28	2.86	2.67	0.36	0.03	0.07	0.19
	After	2.96	3.06	4.40	-0.02	0.03	0.03	0.06
**Pop. density**	Before	0.48	0.05	0.22	0.86	0.22	0.22	0.43
**(6 km^2)**	After	0.32	0.31	0.46	0.02	0.00	0.00	0.01
**Travel time (h)**	Before	21.23	42.18	19.26	-1.51	0.17	0.22	0.54
	After	26.15	27.31	17.00	-0.08	0.03	0.04	0.11
**Towns (km)**	Before	88.80	86.68	34.39	0.04	0.06	0.07	0.13
	After	76.12	76.32	41.09	0.00	0.04	0.04	0.11
**Wet months**	Before	9.81	9.27	1.90	0.27	0.02	0.05	0.17
	After	9.76	9.68	1.97	0.04	0.03	0.06	0.10
**Pop. density**	Before	0.84	0.17	0.38	1.84	0.33	0.34	0.67
**(12 km^2)**	After	0.75	0.72	0.45	0.09	0.02	0.02	0.03
**Ecoregion 1**	Before	0.00	0.00	0.05	-	0.00	0.00	0.00
**Puna**	After	0.00	0.00	0.01	-	0.00	0.00	0.00
**Ecoregion 2**	Before	0.20	0.01	0.11	0.47	0.09	0.09	0.19
**Yungas**	After	0.07	0.06	0.24	0.01	0.00	0.00	0.00

* eCDF Med/Mean/Max: Median/Mean/Maximum differences in empirical cumulative distribution functions.

Supplementary Table S15. Covariate balance before and after matching one cohort of data points in Indigenous Territories (IT, n=95,713) and mining concessions (n=500,000). Successfully matched: n=9,971.

		Mean IT	Mean Control	SD Control	Std mean difference	eCDF Med *	eCDF Mean *	eCDF Max *
**PS distance**	Before	0.88	0.02	0.08	3.78	0.49	0.48	0.94
	After	0.35	0.33	0.33	0.10	0.03	0.04	0.13
**Road (km)**	Before	55.29	9.01	8.07	0.76	0.48	0.44	0.61
	After	25.00	21.46	21.83	0.06	0.04	0.05	0.13
**Settlement**	Before	4.28	3.61	2.30	0.20	0.05	0.05	0.10
**(km)**	After	3.56	3.50	2.56	0.02	0.02	0.02	0.06
**Forest edge**	Before	0.61	0.50	0.80	0.14	0.00	0.01	0.14
**(km)**	After	0.63	0.43	1.13	0.25	0.03	0.06	0.23
**Rivers (km)**	Before	9.55	8.78	12.73	0.07	0.03	0.04	0.20
	After	5.38	6.96	12.93	-0.15	0.10	0.13	0.34
**Slope**	Before	5.28	7.05	9.25	-0.27	0.04	0.04	0.06
	After	5.39	6.94	8.93	-0.24	0.06	0.07	0.13
**Pop. density**	Before	0.48	0.56	0.50	-0.16	0.04	0.04	0.08
**(6 km^2)**	After	0.55	0.56	0.50	-0.02	0.00	0.00	0.01
**Travel time (h)**	Before	21.23	8.97	4.70	0.88	0.92	0.17	0.56
	After	13.27	13.84	11.06	-0.04	0.05	0.05	0.13
**Towns (km)**	Before	88.80	86.31	33.57	0.05	0.10	0.10	0.18
	After	70.01	69.90	40.23	0.00	0.01	0.11	0.31
**Wet months**	Before	9.81	11.23	1.75	-0.72	0.03	0.014	0.41
	After	10.48	10.71	2.06	-0.12	0.03	0.04	0.11
**Elevation (m)**	Before	352	484	501	-0.43	0.02	0.04	0.34
	After	382	414	438#	-0.10	0.01	0.02	0.15
**Rainfall (mm)**	Before	2162	3639	1090	-3.04	0.36	0.35	0.73
	After	2536	2700	1026	-0.34	0.04	0.05	0.19
**Ecoregion**	Before	0.00	0.02	0.14	-	0.01	0.01	0.02
**Puna**	After	0.00	0.01	0.08	-	0.00	0.00	0.01
**Ecoregion**	Before	0.20	0.17	0.37	0.08	0.02	0.02	0.03
**Yungas**	After	0.16	0.23	0.42	-0.18	0.04	0.04	0.07

* eCDF Med/Mean/Max: Median/Mean/Maximum differences in empirical cumulative distribution functions.
